# Mapping nanocrystal orientations via scanning Laue diffraction microscopy for multi-peak Bragg coherent diffraction imaging

**DOI:** 10.1107/S160057752300365X

**Published:** 2023-05-30

**Authors:** Yueheng Zhang, J. Nicholas Porter, Matthew J. Wilkin, Ross Harder, Wonsuk Cha, Robert M. Suter, He Liu, Landon Schnebly, Richard L. Sandberg, Joshua A. Miller, Jon Tischler, Anastasios Pateras, Anthony D. Rollett

**Affiliations:** a Department of Materials Science and Engineering, Carnegie Mellon University, Pittsburgh, PA 15213, USA; b Department of Physics and Astronomy, Brigham Young University, Provo, UT 84602, USA; c Advanced Photon Source, Argonne National Laboratory, Lemont, IL 60439, USA; d Department of Physics, Carnegie Mellon University, Pittsburgh, PA 15213, USA; University of Tokyo, Japan

**Keywords:** Laue diffraction, BCDI, synchrotron, Bragg diffraction

## Abstract

The recent commissioning of a movable monochromator at the 34-ID-C endstation of the Advanced Photon Source has vastly simplified the collection of Bragg coherent diffraction imaging (BCDI) data from multiple Bragg peaks of sub-micrometre scale samples. Laue patterns arising from the scattering of a polychromatic beam by arbitrarily oriented nanocrystals permit their crystal orientations to be computed, which are then used for locating and collecting several non-co-linear Bragg reflections. The volumetric six-component strain tensor is then constructed by combining the projected displacement fields that are imaged using each of the measured reflections via iterative phase retrieval algorithms. Complications arise when the sample is heterogeneous in composition and/or when multiple grains of a given lattice structure are simultaneously illuminated by the polychromatic beam. Here, a workflow is established for orienting and mapping nanocrystals on a substrate of a different material using scanning Laue diffraction microscopy. The capabilities of the developed algorithms and procedures with both synthetic and experimental data are demonstrated. The robustness is verified by comparing experimental texture maps obtained with Laue diffraction microscopy at the beamline with maps obtained from electron back-scattering diffraction measurements on the same patch of gold nanocrystals. Such tools provide reliable indexing for both isolated and densely distributed nanocrystals, which are challenging to image in three dimensions with other techniques.

## Abstract 2

A workflow for orienting and mapping nanocrystals on a substrate of a different material lattice using scanning Laue diffraction microscopy has been established.

## 1. Introduction

Understanding the relationship between macroscopic mechanical properties of materials and their nanoscale structure is a long-term endeavor. Theoretical models of phenomena such as twin formation and dislocation motion require three-dimensional (3D) experimental validation at the nanoscale (Billinge & Levin, 2007 ▸; Meyers & Chawla, 2008 ▸; Erik van der Giessen *et al.*, 2020 ▸; Bechthold & Weaver, 2017 ▸ **)**. Bragg coherent X-ray diffraction imaging (BCDI) has emerged as a strong candidate for such experiments. This non-destructive technique provides 3D images of lattice strain fields in nanocrystals with resolution approaching sub-10 nm and even allows *operando* imaging of defects (Ulvestad *et al.*, 2015 ▸). Upgrades to X-ray light sources in the coming years will enable BCDI at sub-nanometre resolution and may enable near atomic resolution of these strain fields (https://www.aps.anl.gov/APS-Upgrade/About-the-APS-Upgrade).

In BCDI, a nanocrystal is illuminated by a monochromatic, coherent X-ray beam. 3D real space images of the crystal electron density are enabled by collecting the 3D reciprocal space intensities of the coherently scattered Bragg peak during a rocking scan measurement. A detector records a series of two-dimensional (2D) slices of the 3D Bragg peak at each angular step in the far-field (Robinson *et al.*, 2001 ▸). The 3D intensity distribution of the coherent X-ray diffraction pattern is then reconstructed via iterative phase retrieval algorithms to yield the real space electron density of the crystal (Fienup, 1982 ▸, 1987 ▸). The real-space electron density is a complex function whose phase contains the projection of the coarse-grained lattice displacement along the scattering vector. To recover the full strain tensor of the crystal at least three BCDI data sets from linearly independent Bragg reflections are needed (Newton *et al.*, 2010 ▸; Hofmann *et al.*, 2017 ▸).

Recent methods for coupled reconstructions from multiple Bragg reflections of the same nanocrystal provide the full strain tensor in every volume element or voxel of the reconstructed volume (Wilkin *et al.*, 2021 ▸; Hofmann *et al.*, 2017 ▸). In previous work published by Newton *et al.* (2010 ▸), knowing the growth direction of a ZnO nanorod and finding one (101) reflection allowed an orientation matrix to be derived and BCDI datasets from six different reflections to be recorded for the first time (Newton *et al.*, 2010 ▸). In the work of Hofmann *et al.* (2017 ▸), in which they measured multiple Bragg peaks from the same isolated nanocrystal, the crystal orientation was determined beforehand using broadband Laue diffraction at another instrument. Yang *et al.* (2022 ▸) demonstrated that the orientation of exposed nanocrystals can be found using electron back-scatter detection (EBSD), which could then be used to align the crystal for BCDI experiments. While that work shows the potential of EBSD to pre-determine the crystal orientation for subsequent BCDI measurements, it only provides orientations of grains whose top surface is accessible for electron diffraction because electron penetration into metals at 70° incidence is limited to a few nanometres. For efficient determination of crystal orientations of buried nanocrystals, or pairs of neighboring grains in a 3D polycrystalline material, one needs a method to determine orientations *in situ*. This is especially true if multi-peak BCDI is to be applied to *in situ* deformation experiments such as tensile or compressive loading where grains may move and rotate making the tracking of multiple Bragg peaks in reciprocal space impossible without repeated Laue orientation mapping at the same beamline.

Laue microscopy is a powerful tool for investigating local crystal structure and microstructural evolution during deformation, thermal expansion and other strained states (Greilinger, 1935 ▸; Huang, 2010 ▸; Gatti, 2015 ▸). Modern developments include differential aperture X-ray microscopy (DAXM) (Larson *et al.*, 2002 ▸) which uses a Pt wire to scan through the diffraction signal near the sample to probe the depth origin of each diffracted beam by correlating blocked reflections with the location of the wire. Additionally, pencil-beam Laue diffraction tomography has been demonstrated using a tomographic approach for the 3D reconstruction (Ferreira Sanchez *et al.*, 2015 ▸). Even though these methods provide elastic strain mapping, they are challenging to combine with BCDI and do not currently reach significantly sub-micrometre spatial resolution in 3D. Combining BCDI with micro-Laue diffraction microscopy provides high spatial and orientation resolution with reasonable acquisition times, *i.e.* using Laue microscopy to determine the orientation of multiple grains in a polycrystal, acquire multiple BCDI peaks from each grain and reconstruct the 3D shapes and strain fields with sub-10 nm resolution. The existing Laue diffraction microscopy at 34-ID-E uses DAXM with ray-tracing analysis to register the resulting diffracted signals to their corresponding depths to achieve depth resolution (Liu & Ice, 2014 ▸). The double-crystal Si (111) monochromator at beamline 34-ID-E enables fast switching between monochromatic and polychromatic modes, with energy tunable between 7 and 30 keV. The sample is scanned using a precise three-axis stage, positioned at a 45° angle to the incoming X-ray beam for a sample depth range of approximately 100 µm. Laue patterns are captured by a Perkin-Elmer pixel-array detector (409.6 mm × 409.6 mm, 2048 × 2048 pixels, 16-bit dynamic range), mounted in a 90° reflection geometry approximately 500 mm above the sample. The detailed experimental geometry can be found in the work of Liu *et al.* (2004 ▸). The resulting overlapping Laue patterns generated within each subgrain volume along the microbeam are reconstructed and indexed using *LaueGo* (written in C and Igor Pro), which was utilized for routine operations of the 3D X-ray Laue diffraction microscope at the E hutch of sector 34-ID (Liu *et al.*, 2004 ▸).

Recently, the 34-ID-C beamline of the Advanced Photon Source integrated a movable monochromator, which allows switching between a monochromatic X-ray beam used for BCDI and a polychromatic (pink) beam used for Laue diffraction. Laue diffraction allows users to determine individual crystal orientations at the beamline in the laboratory frame and has greatly simplified multi-reflection BCDI at 34-ID-C (Pateras *et al.*, 2020 ▸).

To acquire an orientation map of many crystals, it is essential to automate indexing methods for scanning Laue diffraction microscopy. Here, we demonstrate such an automated workflow that executes the required Laue diffraction analysis, taking a scanning Laue diffraction dataset and returning an orientation map of the sample akin to EBSD. This technique can be used to rapidly select crystals for BCDI measurement, increasing the potential experimental throughput while at a beamline.

To extend the Laue orientation methods to polycrystalline samples and acquire an orientation map in a fashion similar to existing Laue diffraction microscopy at 34-ID-E (Liu & Ice, 2014 ▸), several tools had to be developed to automate the Laue analysis for the measurements performed at 34-ID-C.

In previous work, we demonstrated Laue diffraction microscopy at 34-ID-C for orienting a single isolated gold nanocrystal (Pateras *et al.*, 2020 ▸). In that work, the software package *LaueGo* was used to index Laue patterns (Liu *et al.*, 2004 ▸). However, the precision of indexing arbitrarily oriented nanocrystals was only 0.2°. This error originated in the calibration of the detector position and orientation. In addition, the procedure of identifying the Bragg peaks that belong to the same single nanocrystal required manually moving the monochromator crystals in and out of the pink X-ray beam. We discuss the various experimental modalities in Section 2.1. To implement a fully automated Laue microscopy capability at 34-ID-C several analysis components had to be developed to deal with the unique features of the Laue diffraction measurement at 34-ID-C. First, the closer proximity between the sample and detector at 34-ID-C (∼25 mm) compared with 34-ID-E (∼500 mm) hinders the use of a scanning differential aperture for depth resolution, thus increasing the difficulties of indexing overlapping Laue diffractions. Second, contrary to 34-ID-E, the photon-counting Eiger2 detector exhibits a very different noise variation than that seen at 34-ID-E.

There are several reasons for the difference in the noise between the Eiger2 and the Perkin-Elmer detector. First, the Perkin-Elmer detector at 34-ID-E is mounted relatively far (500 mm) from the beam/sample interaction point. Therefore, the non-crystallographic scattering is nearly uniform across the detector face. Additionally, the Perkin-Elmer is not photon counting and displays significantly less variation in detected photon density across the detector surface. 34-ID-E uses a simple threshold to isolate peaks from the near uniform background on the Perkin-Elmer. At 34-ID-C the photon-counting detector (Eiger2) is much closer to the sample (∼67 mm). The background scattering is therefore non-homogeneous across the detector. Near the center of the detector the background is nearly 1000 photons s^−1^ whereas at the edges it drops down to around 400 photons s^−1^. The dimmer peaks in the Laue pattern can be as low as 400 photons s^−1^. Therefore, a simple threshold does not work, because the dimmer peaks near the edges would be eliminated by the higher threshold value. This motivates the effort to subtract a spatially variable background. At 34-ID-E the standard workflow is designed for samples composed of a single material. At 34-ID-C (*i.e.* this work) two materials are typically present, *i.e.* nanocrystals atop a crystalline substrate. Typical combinations are silicon or SrTiO_3_ (STO) for the substrate with gold or platinum nanocrystals. The standard workflow at 34-ID-E is not intended for indexing Laue patterns with multiple materials simultaneously, especially not where the phase of interest has far weaker peaks than the substrate. The 34-ID-C workflow segments all the peaks, separates out the substrate peaks from those of the nanocrystals and indexes them separately. Additionally, we learned quickly that the movable monochromator implemented for this project did not have a reliable energy calibration curve. As a result, the detector calibration methods developed at 34-ID-E could not be used as-is to refine the relevant parameters (Barabash & Ice, 2014 ▸). Thus, developing a new workflow was required for detector calibration, background subtraction and automatic peak identification in the recorded Laue patterns.

## 2. Scanning Laue diffraction microscopy

### 2.1. Experimental modalities

**Figure 1:**
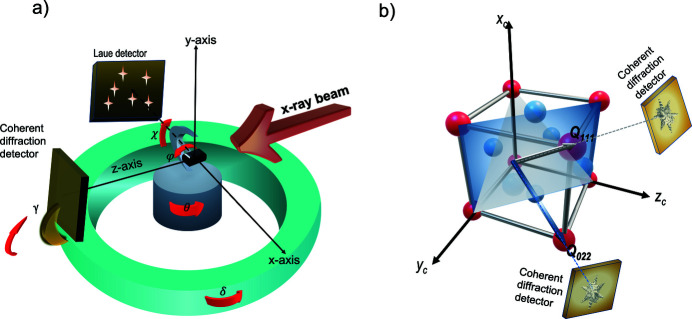
(*a*) Schematic of the experimental geometry at the 34-ID-C beamline. Rotations in (*a*) are shown at one diffractometer snapshot; when the sample stage is rotated to θ = 0°, φ = 0° and χ = 90° and the BCDI detector is rotated to γ = 0°, δ = 0°. At θ = 0°, the incident beam is perpendicular to the detector surface when the detector is sitting at δ = 0° and γ = 0°. At χ = 90° and φ = 0°, the surface normal of the sample is parallel to the *y*-axis. The diffractometer is as specified in the *Spec Sixc* mode (Vlieg, 1997 ▸). The incident X-ray beam is antiparallel to the *z*-axis of the laboratory frame. The Laue detector is shown with its surface being normal to the *x*-axis of the laboratory frame; the *x*-axis points towards the outboard of the synchrotron. The BCDI detector sits at the end of an arm and has three degrees of freedom, the detector distance and the angles γ, δ, as shown. The origin is located at the center of rotation of the diffractometer within a couple of micrometres. (*b*) Schematic of the two Bragg peak measurement for the detector calibration. The two *Q* vectors correspond to two separate measurements. For the first measurement the crystal is oriented so that the monochromatic beam diffracts from the (111) atomic planes. The intensity of the (111) Bragg peak, which is recorded by the BCDI detector, is optimized by tuning the θ, φ, χ angles of the sample stage. Then a Laue pattern is collected at the exact position after switching to the pink beam. The same process is repeated for measuring the 022 Bragg peak and the corresponding Laue pattern at the given position. All coordinate systems are right-handed.

A schematic of the experimental geometry is shown in Fig. 1 ▸(*a*). The sample is placed on top of a piezo stage with three degrees of freedom of rotation (angles θ, φ, χ) and three degrees of freedom for translation (axes *x*, *y*, *z*). Two different detectors are used for recording Laue patterns and coherent diffraction patterns. The Laue detector (Eiger2) is aligned such that its front surface is facing outboard of the synchrotron from the inboard side of the beam. This detector can move in *y* up or down vertically and in *x* inboard/outboard away/towards from the sample but is fixed in *z*. The detector used for collecting BCDI datasets is attached to the diffractometer arm and has three degrees of freedom. The distance of this detector from the sample can be varied within the range 0.5–2.5 m while the arm allows rotation about the *x* and *y* axes, angles γ and δ, respectively, as defined in Fig. 1 ▸(*a*). With a prior knowledge of the energy bandwidth in the beam and symmetry of the lattice, the measured diffraction angles can be used to calculate the orientation of the diffracting lattice to an accuracy of 0.01° (Larson & Levine, 2013 ▸).

When a beam with finite spectral bandwidth illuminates a single crystal, every reciprocal lattice point lying within the shell bounded by the Ewald spheres of the energy bandwidth of the beam will simultaneously be excited. For a 300 nm gold nanocrystal, one may expect four to six reflections on the current Eiger2 500k detector (1024 × 512 pixels, 75 µm × 75 µm pixel size) given the current experimental geometry. In the current experimental configuration, the distance between the detector and focus is around 25 mm. The detector covers a solid angle Ω = *A*/*d* ^2^ = 1024 × 512 × (75 µm)^2^/(25 mm)^2^ ≃ 4.7 sterad.

In a scanning Laue microscopy measurement, a sample containing many crystals is scanned across a broadband beam. For the procedure detailed here, de-wetted gold nanocrystals were grown on a (100) oriented niobium-doped, strontium titanate substrate (0.05 wt% Nb:STO from MSE Supplies). At 34-ID-C, the pink beam energy bandwidth is approximately 5–20 keV (0.8–2.5 Å). The slitted, unfocused X-ray beam is 30 µm × 70 µm. The beam is focused by a set of KB mirrors down to a beam spot of 0.5 µm × 0.7 µm. For the collection of BCDI patterns a Lynx T1 detector with GaAs sensor is used which has a pixel pitch of 55 µm and an orthogonal 512 × 512 grid.

### 2.2. Laue detector calibration

Calibrating the Laue detector geometry is crucial for accuracy and reliability of the determined orientations. For accurately indexing the measured Laue patterns from arbitrarily oriented nanocrystals, it is necessary to determine the detector orientation relative to the laboratory frame to within tens of milliradians or less. Following the implementation used in *LaueGo* the detector is defined by a rotation vector **R** and translation vector **P**, each having three components. Traditionally, the Laue detector calibration at the neighboring 34-ID-E endstation consists of two steps. The first step requires recording a Laue pattern from a single-crystal silicon wafer with known crystallographic orientation and indexing it with *LaueGo* based on estimated **R** and **P** vectors. During the second step, the energy of at least three Laue peaks, seen in the previously recorded Laue pattern, are determined with an accuracy of less than 1 eV by scanning the energy of the X-ray beam. Knowing the energies of the peaks and the lattice constant of silicon, one can determine the orientation of the lattice with high certainty.

At 34-ID-C, the monochromator used for these experiments is not accurate enough to enable this calibration method. Therefore, determining the energy of several peaks with sufficient accuracy is not possible. Additionally, scanning the energy for each of the peaks can be time consuming. However, at 34-ID-C we can determine the 2θ value of a single found monochromatic Bragg peak with high accuracy using the diffractometer. This allows an accurate knowledge of a single X-ray energy and the subsequent determination of the sample orientation when a second Bragg reflection is identified. The procedure can be conducted with a silicon (001) oriented substrate where locating two or more Bragg peaks from the substrate is not difficult. More specifically, the (022) Bragg reflection for silicon is found and the goniometer angles recorded at the position of maximum intensity. This allows the determination of the **Q**_022_ diffraction vector simultaneously in the laboratory and crystal frames. By repeating the above steps for a (111) silicon reflection, a second orientation vector is determined in both laboratory and crystal reference frames. The two vectors are depicted in Fig. 1 ▸(*b*) and can be expressed in the laboratory and crystal frames through a transformation of axes as

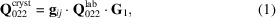




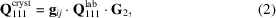




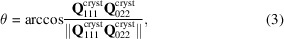

where 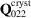 is the **Q**_022_ diffraction vector in the crystal frame and 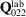 is the **Q**_022_ diffraction vector in the laboratory frame. Similarly, 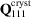 and 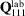 are, respectively, the **Q**_111_ diffraction vector in the crystal frame and laboratory frame. **g**_*ij*_ is an orientation matrix for the crystal with respect to the sample reference frame. **G**_*i*_ is a similar rotation matrix but for the sample with respect to the laboratory frame. Then, orthonormal vector bases are constructed in the laboratory and crystal reference frames and the matrices are derived to transform a vector expressed in the crystal reference frame to the laboratory frame (see Section S1 in the supporting information). Using the known sample orientation and initial guess of **P** and **R**, a Laue pattern can be simulated for any arbitrary sample orientation at which a Laue pattern is measured. This is typically done when the sample stage is set to θ = 0°, φ = 0°, χ = 90°. At these settings, the incident beam is parallel to the surface of the sample. Measurements are taken when the sample is at an angle of incidence of 10°, for the beam to scatter from the sample and diffract to the detector. Note that a pair of the monochromatic beam Bragg peaks are required [*e.g.* one for the (111) and one for the (022) reflections] and at least one pink beam Laue pattern. Then multivariable optimization algorithms are used to refine the values of the **P** and **R** vectors to match the simulated Laue pattern to the measured one (see Section S3 in the supporting information).

The translation vector **P** defines the distance between the center of the detector surface and the origin in the laboratory frame where the beam illuminates the sample, as shown in Fig. 1 ▸(*a*). The Rodrigues rotation vector **R** rotates the detector surface from the origin to its actual location. The values of each component of the **P** vector are estimated initially by measuring the horizontal and vertical offsets of the center of the surface of the Laue detector from the origin with a ruler. Each of the aforementioned components of **P** is determined with a couple of millimetres accuracy and formed the initial guess of the translation vector **P** = [0, −25, −65] mm. The rotation vector **R** is estimated by determining the rotation matrix and the Rodrigues rotation formula (see Section S3). We assign the initial values of the rotation vector **R** = [2.3, 0.0, 2.2] rad, indicating that the detector is placed on the inboard side of the sample facing straight outboard.

The above pair of vectors is optimized by combining the Nelder–Mead algorithm (Nelder & Mead, 1965 ▸) with a grid search method. For the definition of the cost function, the Laue patterns are simulated by the forward model, compared with the experimental data, and the distances are calculated between the extracted peaks in the experimental data and corresponding nearest peak positions in the forward modeled Laue patterns. We define as loss the mean of the absolute values of distances between predicted and experimentally determined Bragg peaks (see Section S9). The termination criterion for the optimization process is set as a maximum error of less than one pixel. When the optimization reached a value below one pixel the following values for a particular experiment (the values vary each time the apparatus is set up) were obtained for the translation and rotation vectors, respectively, **P** = [−0.653, −25.946, −68.220] mm and **R** = [2.23, −0.009, 2.205] rad for this work.

Although Nelder–Mead often optimizes the objective function within tens of iterations, it can be trapped in local minima. Accordingly, we varied the initial values for the optimization. More specifically, both **P** and **R** have three independent components. The initial value of *P*_1_ (the first component of **P**) can vary from −5 to 5 mm in steps of 2. Similarly, the initial value of *P*_2_ varies from −20 to −30 mm and *P*_3_ varies from −60 to −70 mm with the same step size as *P*_1_. The initial value of all components in **R** varies from 0 to 5 rad with a step size of 1 rad. The final values obtained from different initial guesses exhibit variability. Section S9 shows a comparison of the outcomes determined from varying initial values of *P*_2_. We were able to find the optimal value of the components of the translation vector **P** and rotation vector **R** with the same procedure. We conclude that combining Nelder–Mead with a grid search method is effective for this optimization task, which is typically performed once per beam time.

The optimized translation vector **P** and rotation vector **R** are used as input parameters for *LaueGo* to index Laue patterns. The other inputs to *LaueGo* include a list of coordinates of the peak positions on the detector image and the crystal lattice parameters. The lattice parameters of a crystalline material can be determined using techniques such as X-ray diffraction or using standard crystal lattice parameters as reported by Couderc *et al.* (1959 ▸).

### 2.3. Image processing

Figure 3 illustrates the overall image processing workflow for scanning Laue diffraction microscopy. The result yields a background-subtracted diffraction pattern with peaks identified from both the substrate and individual nanocrystals. These patterns are then used to calculate a list of orientations and their corresponding spatial positions in the beamline reference frame. For the collection of Laue patterns, the same experimental geometry was used as reported by Pateras *et al.* (2020 ▸).

**Figure 2:**
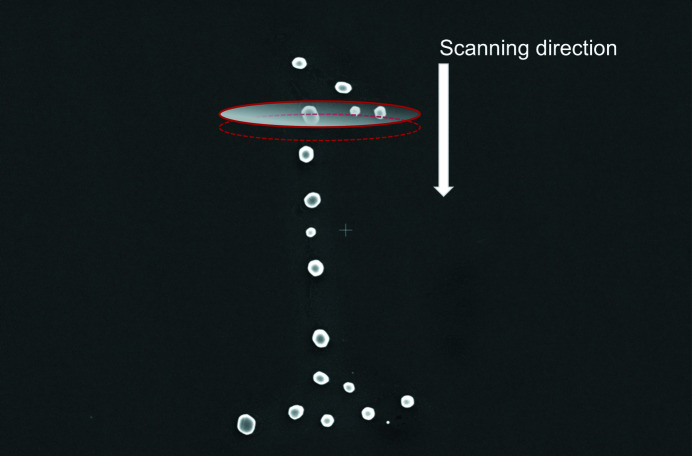
Schematic of the scanning for pink beam across a cluster of nanocrystals on the STO substrate. The X-ray beam (approximate footprint shown in red) is impinging on three crystals and moves along the direction of the white arrow. A 2D mesh scan is performed over a total area of 30 µm × 2 µm using a step size of 250 nm and 500 nm for the vertical and horizontal directions, respectively. This ensures that all nanocrystals in the cluster are illuminated.

**Figure 3:**
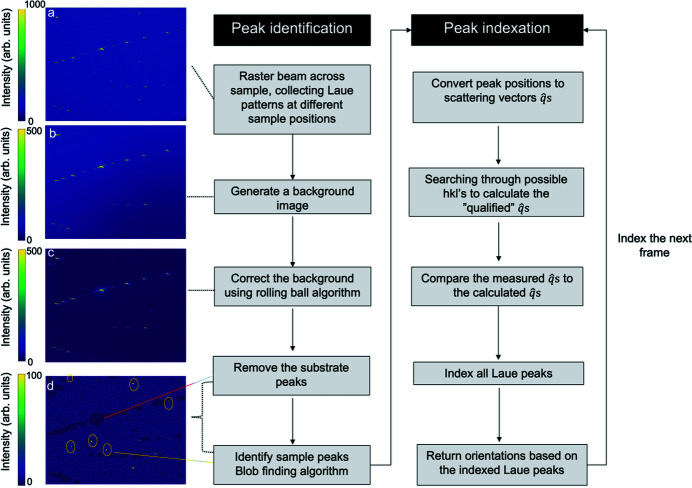
Image processing workflow of the scanning Laue diffraction microscopy method including data collection, and automated data analysis. Panel (*a*) is an example of a diffraction pattern measured on an Au crystal. Panel (*b*) shows the background calculated from the pixel-wise median value of the intensity. Panel (*c*) shows the background corrected by applying the rolling ball algorithm to the background. Panel (*d*) shows the removed substrate peaks denoted by the red line, which correspond to the zero-intensity regions, previously visible as bright peaks in images (*a*)–(*c*). In addition, the identified Au peaks are highlighted with a yellow outline.

We used the pink beam to scan patches of de-wetted, arbitrarily oriented gold nanoparticles on an STO substrate. Each spatial mesh scan was executed using sub-micrometre steps to create redundancy in the data with significant spatial overlap from step to step, shown in Fig. 2 ▸. At each step in the scan, a Laue image such as the one shown in Fig. 3 ▸(*a*) is collected, containing peaks from all nanoparticles illuminated by the beam at that step as well as those from the substrate. Because the sample is at a relatively small incidence angle (∼10°) the beam footprint on the sample results in greater uncertainty in the position parallel to the direction of elongation.

The next step is to locate the positions of all Laue peaks in the raw data to use for indexing. Several factors complicate image segmentation in these Laue datasets, including the presence of high-intensity substrate peaks, variation in flux across the energy profile of the beam, and diffuse scattering. In particular, the small size of the nanocrystals relative to the substrate makes their peak intensities orders of magnitude lower than the substrate peaks. Furthermore, the energy spectrum of the beam is not uniform, such that reflections fulfilling the Laue criterion at energies with low flux cannot be distinguished from the background.

#### 2.3.1. Background subtraction

The indexing program can only index Laue peaks from one material at a time, so it is necessary to first remove the substrate peaks from every image so that only peaks originating from the nanocrystal remain. Because the substrate is a single-crystal STO wafer, its Laue peaks are practically identical in every image of the mesh scan. Global thresholding does not suffice for segmentation of these high-intensity substrate peaks because non-crystallographic scattering by the nanocrystals on the substrate leads to a strongly varying background. The intensities of some substrate peaks with low flux are on the same scale as the background noise in some regions of the detector.

Because the substrate peaks exist in every detector image, they can be considered as part of the background. This background can be determined by calculating the pixel-wise median value of the intensities over all images. The variation in the number of diffracting nanocrystals from frame to frame creates variability in the background intensity, meaning a dataset-wide median background is not sufficient. Nevertheless, this background can be used to locate the substrate peaks. The rolling-ball algorithm (RBA) is used to correct the background before applying a global threshold (Rodrigues & Militzer, 2020 ▸) to segment out the substrate peaks. The RBA can be visualized in the following way. Consider the 2D intensity profile as a 3D surface, where the surface height at each pixel is decided by its intensity. The RBA determines the background by rolling a sphere of fixed radius across the surface and recording its displacement at every pixel. This displacement surface is the background. Subtracting this surface flattens the background, allowing the peaks to be segmented by a simple global threshold.

**Figure 4:**
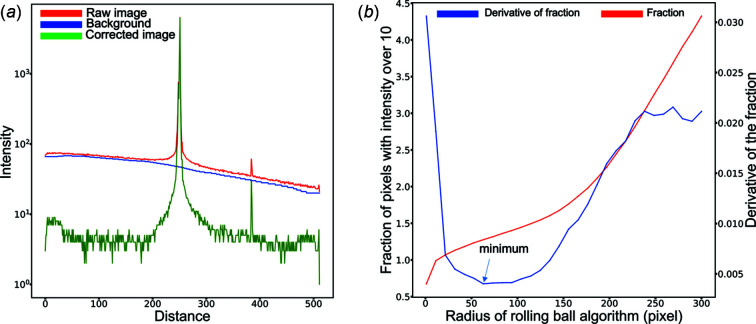
(*a*) Intensity profile of a vertical line of pixels across the detector image [see also Fig. 3 ▸(*a*)] before and after using the rolling ball algorithm. The *x*-axis represents pixels with different distance to the upper pixel of the line on the detector. The *y*-axis represents the intensity values of the corresponding pixels. The red solid line corresponds to the raw image, the blue solid line corresponds to the resulting background, and the green line corresponds to the corrected image. (*b*) The fraction of pixels whose intensity is larger than the threshold value and the first derivative of the fraction of pixels with respect to the tested radius (the parameter of the rolling ball algorithm *r*) plotted as a function of radius.

Compared with other approaches like local adaptive thresholding (Chow & Kaneko, 1972 ▸; Sauvola & Pietikäinen, 2000 ▸; Gonzalez & Woods, 2009 ▸) and automatic thresholding (Otsu, 1979 ▸; Kapur *et al.*, 1985 ▸; Sezan, 1990 ▸), the RBA needs only one user input parameter (the radius of the 3D ball) and is computationally efficient (Sternberg, 1983 ▸, 1986 ▸). As illustrated in the 1D intensity profile in Fig. 4 ▸(*a*), the intensity variation in the raw image is large and the average intensity in the left region of the line profile is much higher than in the right region. For instance, the intensity of a peak (distance ≅ 390) is lower than the background in the region near the origin (distance = 0), which makes it indistinguishable from the background using a global threshold. Thus, it is difficult to use simple thresholding to find the peak.

The resulting background (blue solid line) depicts the intensity distribution of the background. The corrected image was generated by subtracting the background obtained from the RBA (blue line) from the raw image (red line). The visibility of the peak seen at *x* ≅ 390 has been improved enough so that it can be distinguished much more easily, simplifying the subsequent peak-finding step.

As mentioned, the ball radius is the only algorithmic parameter in RBA but its value depends on the background distribution found in each experimentally acquired image. To reduce the number of manually input parameters, we used the twiddle algorithm in Section S4 (Thoma, 2014 ▸) to find a radius that minimizes the derivative of the fraction of pixels whose intensities are below a threshold value in the corrected image (raw image with background subtracted) with respect to the tested radius [see Fig. 4 ▸(*b*); details can be found in Section S8]. With the background removed, the images are then passed through a global threshold to remove any remaining noise (Rodrigues & Militzer, 2020 ▸). Using a cluster identification algorithm (Virtanen *et al.*, 2020 ▸), the location of all intensity clusters (substrate peaks) was identified.

#### 2.3.2. Nanocrystal peak identification

**Figure 5:**
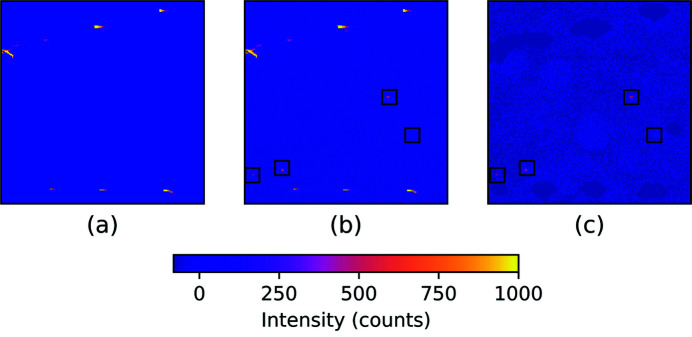
(*a*) A sub-region of the background image [see also Fig. 3 ▸(*c*)]. (*b*) The same sub-region of a raw image before the subtraction of background [see also Fig. 3 ▸(*a*)]. (*c*) The same sub-region of a raw image after the subtraction of background [see also Fig. 3 ▸(*d*)].

The next step is to calculate the background and remove the aforementioned intensity clusters from each image in the raw dataset. First, the image is normalized and a Gaussian filter with σ = 20 is applied to blur the intensities. A median filter is then applied to identify the background for every pixel. Finally, the substrate peaks found in the previous step are subtracted from all raw images. The substrate peaks are enlarged via binary dilation to ensure that intensity fluctuations from frame-to-frame around each peak are fully removed. Fig. 5 ▸ shows the image before and after the subtraction of bright peaks. The black boxes show where the nanocrystal peaks are.

After background subtraction of the images, the peaks belonging to the nanocrystal sample are extracted from each image. The threshold value for each reduced image is determined by Otsu’s approach (Otsu, 1979 ▸). This method calculates the histogram for an image and determines a threshold by minimizing intra-class intensity variance. This single intensity threshold separates pixels into two classes – objects and background. The cluster finding tool is used again to locate the peak positions. Though the rolling ball method identifies the vast majority of substrate peaks, there remains a possibility that some dimmer substrate peaks were missed, causing them to be misidentified as nanocrystal peaks. To mitigate this, the orientation of the substrate is used to forward model all potential substrate Laue peaks. Any forward-modeled peaks found within five pixels of a potential substrate peak are then eliminated from the set of nanocrystal peaks.

### 2.4. Indexing

The indexing module is that used in the *LaueGo* package, developed at 34-ID-E (Liu *et al.*, 2004 ▸). The peak positions on the detector were recorded in millimetres (mm) and are represented by the variable *r*_l_. The center of the detector is the origin [*r*_l_ = (0, 0, 0)], *X* pixels increasing along the *x* direction, *Y* pixels increasing along the *y* direction. Using the peak positions *r*_l_ on the detector, the detector translation and rotation vectors **P** and **R** (determined by Laue detector calibration), respectively, and the crystallographic lattice parameters of the sample, the unitary scattering vector **g** of a Bragg peak is given by **g** = **R**(*r*_l_ + **P**). The output of the indexing routine is the orientation matrix, whose columns consist of the reciprocal lattice vectors of the crystal. The reciprocal lattice vectors can be directly used to identify and locate all potential Bragg peaks that are accessible to our detector and where BCDI datasets can be measured. The indexing routine is summarized below:

(i) Convert all positions of identified peaks from pixel indices to reciprocal space vectors **q** in the laboratory reference frame.

(ii) Use the **q** vectors as input for *Euler* (the indexing program in the *LaueGo* package), along with a few threshold values (Liu *et al.*, 2004 ▸). *Euler* searches through possible **G**_*hkl*_ vectors and calculates the corresponding **q**_s_ (the set of simulated **q** vectors by *Euler*). In this process, several threshold values are used to filter out unlikely calculated **q**_s_. First, a maximum energy is chosen to use for searching, which limits how big the **G**_*hkl*_ can get. Second and third is a central **G**_*hkl*_, and cone angle. This allows the user to tell the program approximately where to look for the **G**_*hkl*_ vectors. In our case, the maximum energy for searching is 24 keV, **G**_001_ is the central **G**_*hkl*_, and the cone angle is 72°, which is the maximum needed for cubic crystals.

(iii) Create possible matching pairs based on the measured **q** and calculated **q**_s_ and compare angles between two measured **q** vectors with the angles between two calculated **q**_s_ vectors. During this process, two threshold values are chosen to determine the possible orientations. The first is a maximum energy to use for matching, which is a larger energy than the maximum energy for searching, that allows the program to identify **G**_*hkl*_ with large energies. In our cases, this energy is 30 keV. The second is an angular tolerance for comparing the angle between two **G**_*hkl*_ vectors with the angle between two **q** vectors. In our cases, the angular tolerance is 0.2°.

(iv) The indexed Laue patterns are used to create a pattern list, which associates each pattern with a location on the sample, orientation matrix, the fitted RMS error of the pattern and the number of matching peaks.

(v) Use the pattern list to calculate the center of mass of each grain. The misorientations between orientations in the pattern list are calculated, then orientations less than an upper limit are considered to be the same grain. Therefore, each grain can include many patterns which appear at different sample-to-beam locations. Finally, the average *x* and *y* positions for the grouped grains are calculated and regarded as the center of mass.

After indexing all Laue patterns, a list is generated, which includes all found orientations from gold nanocrystals and the corresponding locations of those crystals on the sample. These orientations are then used to calculate the positions of the Bragg peaks in the laboratory frame of the diffractometer. Then, a set of two orientation vectors are calculated for each grain, the in-plane vector **uvw** and out-of-plane vector **hkl**, which are expressed in 3D-space Cartesian coordinates (Pateras *et al.*, 2020 ▸). Using the in- and out-of-plane vectors, an experiment-management software such as *Spec* (Swislow, 1996 ▸) is used to calculate all the Bragg peak locations for each indexed crystal, facilitating the multi-peak BCDI dataset collection. The displacement between the pink beam and monochromatic beam on the sample when switching from Laue to BCDI mode is less than 1 µm – see the details of the monochromator design in a paper by Liu *et al.* (2011 ▸) and our implementation in the paper by Pateras *et al.* (2020 ▸). To determine the displacement between the monochromatic and pink beam, we used a CdWO_4_ wafer, which luminesces at the X-ray beam footprint locations.

## 3. Validation

### 3.1. Sample and experimental configuration

The samples consist of de-wetted gold crystals on a (100)-oriented, 0.05 wt% niobium-doped, STO substrate (MSE Supplies). Standard optical lithography was performed with AZ330 photoresist and then developed to provide distinct patches of isolated gold nanocrystals. This facilitated locating specific nanocrystals in EBSD and with the optical microscope at 34-ID-C (Beitra *et al.*, 2010 ▸). Gold was then deposited to 30 nm thickness via thermal evaporation. The final lift-off of the remaining resist was performed in 1-methyl-2-pyrrolidinone (NMP) solution with sonication for 10 min. Afterwards, the sample was rinsed with acetone and deionized water. The sample was then annealed in a furnace at 900°C for 16 h in air and allowed to cool down slowly to room temperature, similar to methods described elsewhere (Kovalenko *et al.*, 2017 ▸). The average crystal size is approximately 0.4 µm, making these crystals ideal for BCDI at APS 34-ID-C. Fig. 2 ▸ shows the lithographically patterned cluster of nanocrystals scanned by the pink beam. The beam size is approximately 0.6 µm × 0.7 µm; however, the tails of the beam can be tens of micrometres in extent but several orders of magnitude dimmer. The scanned area was roughly 30 µm × 2 µm. A 2D scan was conducted with step sizes of 500 nm along the *x*_lab_ (horizontal) direction and 250 nm along the *z*_lab_ (vertical) direction.

### 3.2. Results and discussion

**Figure 6:**
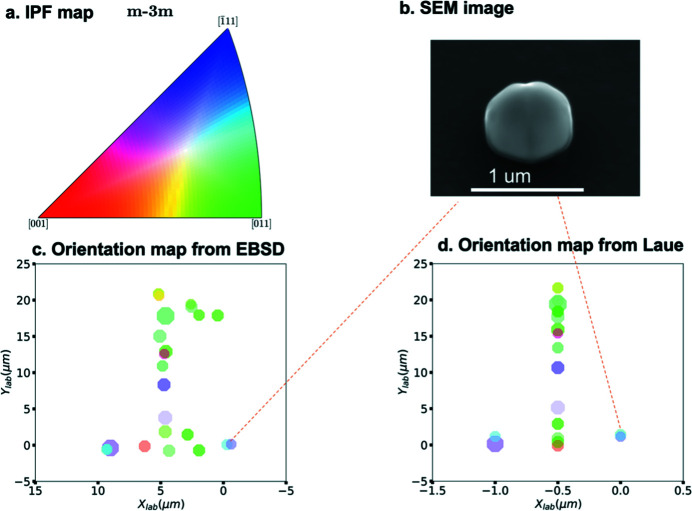
(*a*) An inverse pole figure (IPF) color map for Au. (*b*) Zoomed image as an example of a twin pair. (*c*) EBSD map with IPF color representing the orientation of 22 crystals in the transverse direction. (*d*) Laue map with IPF color representing the orientation of 19 crystal in the transverse direction.

The automatic indexing method was applied to the experimentally measured data and a grain map was returned, which can be seen in Fig. 6 ▸. In summary, 19 crystals were indexed from their X-ray Laue patterns. The misorientations of each matching pair between EBSD and Laue results were calculated and are shown in Table 1 ▸. From the Laue results, 19 indexed out of 22 nanocrystals are within 19° misorientation to the mean orientation of corresponding crystals in EBSD; 13 nanocrystals are within 10° and 8 nanocrystals are within 6°.

**Figure 7:**
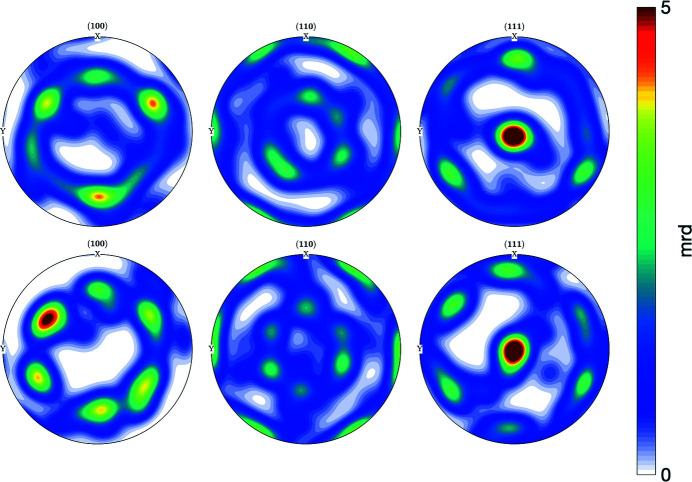
Pole figures for the 22 gold nanocrystals from EBSD experiment (top) and 19 Laue results (bottom).

To illustrate the results quantitatively, we conducted a texture analysis from both EBSD and Laue diffraction measurements. According to the pole figures (PFs) from both results shown in Fig. 7 ▸, the textures are similar to each other in all the PFs. First, nearly all of the crystals have (111) planes normal to the surface of the substrate. Furthermore, we observe an in-plane texture with approximately hexagonal symmetry, likely a consequence of the STO substrate’s impact on the preferred alignment of the gold crystals (Kovalenko *et al.*, 2017 ▸). Fig. 7 ▸ shows that {111}〈110〉 texture components exist among the nanocrystals in both experiments indicating that the results from the Laue analysis match well with the EBSD results.

The constructed 2D Laue map shows 19 grains with less than 19° misorientation for cases where the same grain could be identified in EBSD and in Laue. The orientation map obtained from EBSD measurements in Fig. 6 ▸(*c*) is compared with the reconstructed 2D orientation map from the Laue indexing result in Fig. 6 ▸(*d*). Figs. 6 ▸(*c*) and 6(*d*) are colored with the inverse pole figure (IPF) color key shown in Fig. 6 ▸(*a*) according to the transverse direction in the plane. As a qualitative estimate of the spatial error of our method, we used a scanning electron microscope (SEM) map as the ground truth and calculated the difference between each matched pair from SEM maps and our method. Because we only scanned a length of 2 µm along the *z*_lab_ direction with a step size of 500 nm, we do not have the spatial resolution to quantify the spatial error in this direction. However, the scan fully covered all the crystals in the cluster within this range. Thus, we chose the large purple grain (crystal ID = 11 in Table 1 ▸) in the bottom left of Fig. 6 ▸(*c*) as the origin and calculated the distance *d*_*i*_ between this grain with each other crystals in the *x*_lab_ direction, which is the vertical direction in the map. This provided a distance vector **d**_EBSD_ = (*d*_1_, *d*_2_,…, *d*_*i*_,…, *d*_19_), where the subscript *i* is the crystal ID. Meanwhile, we also set the corresponding grain in Fig. 6 ▸(*d*) as the origin and calculated the distance of each other crystal in the *x*_lab_ direction in the Laue map. The distance vector **d**_Laue_ = (*d*_1_, *d*_2_,…, *d*_*i*_,… *d*_19_) is calculated by the same procedure as for **d**_EBSD_. The spatial error is the difference between **d**_EBSD_ and **d**_Laue_ and the result is shown in Table 1 ▸. As mentioned in Section 3.1, the tail of the beam is 5 µm. Thus, the absolute value of the spatial error close to or less than 2 µm is precise enough for us to locate the crystals for further BCDI experiments.

There are five reasons for the large misorientations for some grains as well as missing pairs of grains. First, the integrated intensity of the Laue diffraction spots of a given grain is proportional to the illuminated volume of that grain (Warren, 1990 ▸). The grain sizes range from 0.2 to 1.2 µm (see Fig. S4 in Section S5), so the intensity of the Laue reflections varies significantly. Thus, when small nanocrystals are illuminated, planes with low structure factors might not provide enough diffracted intensity, therefore peaks from such crystals are difficult to identify.

Second, error can also arise from nanocrystals rotating and drifting during illumination by high-energy X-rays. Our samples differ from those in the cited paper by Yang *et al.* (2022 ▸) which are de-wetted Fe–Ni microcrystals on single-crystal sapphire, so their experience with misorientation determination is not necessarily transferable to ours.

The third reason is that the orientation obtained from EBSD is sensitive to (among many other factors) the local surface normal (Nowell *et al.*, 2005 ▸). In a polished polycrystalline sample, this can be assumed to be parallel to the normal to the sample surface, but for the quasi-hemispherical nanocrystals shown in Figs. 2 ▸ and 6 ▸(*b*) there is no guarantee of that. The high-resolution SEM images for crystals (see Fig. S6 in Section S7) show that most crystals are faceted. With such large surface tilts for those highly faceted crystals, it is difficult to index the grains with high confidence using EBSD (Nowell *et al.*, 2005 ▸).

The fourth reason is that EBSD mounting can cause the misorientations errors. The mount which was used for both EBSD and the beamline was a simple aluminium SEM stub to which the sample was attached with double-sided carbon tape. The tilt angle used in EBSD analysis is nominally 70°, but we did not calibrate this angle for the measurement. The net result is that there can easily be a few degrees offset between the orientation reported by the EBSD measurement and the true orientation. Therefore, less than 19° of misorientation is acceptable when we qualitatively match our Laue index results with EBSD results.

The last reason is the inability of *LaueGo* to reliably index overlapping Laue patterns. For example, when more than four grains are illuminated and there are five or fewer identified peaks for each grain, *LaueGo* is likely to return only a subset of correct orientations and/or the false-positive orientations. However, *LaueGo* does provide the output orientation for each grain along with the corresponding RMS error and the number of matched peaks from the experimental data and forward-modeled data. We explored the RMS error and the number of matched peaks for each orientation. The results from the correctly indexed crystals are shown in Table 1 ▸. The highest RMS error is 0.08, which is smaller than those of most false-positive orientations (RMS error ≤ 0.2). The number of matched peaks is another good indicator of the reliability of the experimentally obtained orientations. When the number of peaks is equal to or greater than four, it is highly likely that the indexing is correct. The fractional value is because the crystal is found among different frames and the average of peaks for the same crystal are calculated. If the same crystal appears more than once in the continuous frames or spatially equivalent frames, the orientation is highly likely to be correct. For example, *LaueGo* only indexed three peaks, using one or a few useful parameters for determining the ‘goodness’ of the orientation result. The RMS error, the average number of indexed peaks, and the number of indexed frames for the matching crystals are shown in Table 1 ▸.

In our approach, the Laue diffraction is likely to be more precise for orientation determination at 34-ID-C than EBSD. This is also evident in the work of Yang *et al.* (2022 ▸) who found that the average angular mismatch between orientation matrices misorientations between the Laue and BCDI is 4°, and that between EBSD and BCDI is 6°. We developed our own Laue diffraction technique which is intended to improve upon that available to Yang *et al.* (2022 ▸). For instance, we improved the detector calibration step (see Section 2.2) and increased the accuracy of the detector positioning parameters, which results in the better accuracy of the indexing results. Despite EBSD measurement being widely accepted in the materials community, it is not necessarily the ground truth for isolated crystals with curved surfaces, as we discuss above.

To validate the accuracy of the Laue measurements we successfully moved the Bragg detector to the calculated positions of certain Bragg peaks from the twin crystals and measured them. The misalignment observed between the orientation determined by Laue diffraction analysis and the actual orientation at which the monochromatic Bragg reflection was found to be less than 0.1°. Thus, we concluded that the micro-Laue diffraction is more accurate compared with EBSD to orient nanocrystals at 34-ID-C, which matches the report by Yang *et al.* (2022 ▸). In order to illustrate the randomness of the misorientations, we show multiple examples of combined PFs of different crystals from EBSD and Laue (see Section S10) and find that the discrepancy of orientations (misorientations) from the two analyses lacks any systematic pattern, *i.e.* there is no systematic offset between the Laue and EBSD measurements of orientation.

Knowing the orientations and corresponding grain locations, the in- and out-of-plane vectors **uvw** and **hkl** (Pateras *et al.*, 2020 ▸) for each grain are calculated. These two vectors are then inserted into *Spec* and the diffractometer is moved to the correct orientation for a desired Bragg peak. The orientations and corresponding grain locations can be used for identifying twin crystals. First, face-centred-cubic Σ3 twin boundaries can be described as a 60° rotation about a (111) crystal axis. Second, the precision of the twin orientation relationship means that a pair of twin-related crystals must be neighboring grains, so the distance between them should be small. All this means that a twin boundary is convenient as a highly precise boundary type that serves as a marker of a pair of adjacent grains. In this particular cluster, we collected multiple peaks from one pair of twinned crystals in a single grain. The reconstruction of this dataset is beyond the scope of this paper.

## 4. Conclusions

We present a workflow for automatic indexing of sets of isolated nanocrystals at the 34-ID-C endstation of the Advanced Photon Source that generates a spatial orientation map that is intended to facilitate subsequent measurement of BCDI datasets. We indexed 19 out of 22 nanocrystals within 19° misorientation to the mean orientation of corresponding crystals in EBSD – 13 nanocrystals are within 10° and 8 are within 6°. Previously, acquiring the orientation for each crystal required manual peak identification and indexing, which takes several hours for data collection and data analysis by hand. We acquired the data and indexed the orientations for a set of isolated nanocrystals within 10 min using our method. The ability to index orientations from isolated crystals is expected to generalize to polycrystalline materials.

## 5. Related literature

The following references, not cited in the main body of the paper, have been cited in the supporting information: Koks (2006 ▸); Ploc (1983 ▸); Randle & Day (1993 ▸); Spendley *et al.* (1962 ▸); Zhang *et al.* (2014 ▸).

## Supplementary Material

Supporting Sections S1 to S10. DOI: 10.1107/S160057752300365X/ay5613sup1.pdf

## Figures and Tables

**Table 1:** Nanocrystals of gold indexed from the Laue diffraction data, along with their misorientation from the corresponding EBSD result

Crystal ID	Misorientation (°)	Spatial error (µm)	RMS	Peaks indexed	Frames indexed	Bunge Euler angles (*ZXZ*)
1	16.7	−0.55	0.06	4.6	11	(33.9, 63.8, 130.5)
2	5.19	−0.52	0.02	4.7	6	(93.4, 55.4, 313.6)
3	8.82	−0.87	0.03	5.0	5	(323.8, 50.7, 141.0)
4	5.77	−0.82	0.07	6.0	3	(212.2, 126.8, 130.7)
5	5.59	0.23	0.03	5.0	2	(11.7, 54.5, 138.1)
6	11.83	1.28	0.03	3.0	1	(195.2, 54.8, 312.0)
7	10.78	1.65	0.02	3.8	6	(25.2, 56.8, 135.5)
8	3.94	1.47	0.06	3.7	6	(309.5, 55.8, 188.7)
9	5.88	1.13	0.06	5.7	7	(106.0, 128.3, 316.0)
10	13.79	0.98	0.07	4.0	2	(319.0, 130.4, 144.0)
11	9.16	0.00	0.01	3.6	7	(94.0, 125.5, 133.7)
12	3.14	0.10	0.04	4.8	14	(314.9, 45.9, 194.1)
13	18.64	−0.31	0.04	7.0	1	(203.3, 55.3, 315.2)
14	11.82	−0.26	0.03	5.7	3	(252.7, 143.5, 313.4)
15	8.91	−1.28	0.02	5.0	2	(332.7, 57.0, 228.7)
16	2.57	−0.80	0.02	5.0	5	(126.4, 18.2, 329.7)
17	6.84	0.63	0.08	4.0	2	(23.1, 115.6, 333)
18	5.54	−0.22	0.01	4.5	6	(163.3, 130.0, 195.9)
19	6.17	0.32	0.04	5.1	11	(334.9, 124.3, 135.4)
