# Supplementary material for: Mapping nanocrystal orientations via scanning Laue diffraction microscopy for multi-peak Bragg coherent diffraction imaging
Source: J Synchrotron Radiat. 2023 May 30;30(Pt 4):796–806. doi: 10.1107/S160057752300365X (PMC10325016; doi:10.1107/S160057752300365X)
Supplement: Supplementary file 1 [file s-30-00796-sup1.pdf]

JOURNAL OF SYNCHROTRON RADIATION

Volume 30 (2023)

Supporting information for article:

Mapping nanocrystal orientations via scanning Laue diffraction microscopy for multi-peak Bragg coherent diffraction imaging

Yueheng Zhang, J. Nicholas Porter, Matthew J. Wilkin, Ross Harder, Wonsuk Cha, Robert M. Suter, He Liu, Landon Schnebly, Richard L. Sandberg, Joshua A. Miller, Jon Tischler, Anastasios Pateras and Anthony D. Rollett

The following document provides supplemental information on the main text. This includes Laue detector calibration using two known Bragg peaks (Appendix A), mathematical determination of the rotation/orientation matrix from a specific set of goniometer angles (Appendix B), detector parameter optimization (Appendix C), demonstration of the twiddle algorithm on parameter optimization for rolling ball algorithm (Appendix D), grain size distribution (Appendix E), an example of using rolling ball algorithm with different radius (Appendix F), and high-resolution SEM images for crystals (Appendix G).

S1. Laue detector calibration using two known Bragg peaks

For the detector calibration we used a [001]-oriented Silicon wafer. The crystal orientation matrix was analytically derived by creating orthogonal vector bases in the crystal, sample, and laboratory frames and measuring two independent Bragg reflections with known Miller indices, which in turn, will define two vectors in the crystal and laboratory frames. An initial list of Miller indices was generated for all the peaks that are predicted to appear on the detector. The coordinates of each peak on the detector are calculated by forward modeling the diffraction patterns in Laue geometry at the beamline's experimental setup (Ploc, 1983). Finally, we optimize the detector parameters, which consist of two three-component Rodrigues rotation and translation vectors (Randle & Day, 1993), by minimizing the average distance of each simulated peak on the detector from the experimentally observed peaks on the measured Laue patterns using a direct-search method used for non-linear problems with multiple variables (Nelder & Mead, 1965; Spendley *et al.*, 1962).

We measured the $(1\bar{1}\bar{1})$ and $(2\bar{2}0)$ Bragg reflections by rotating the sample to specific θ, χ, ϕ angles determined in the laboratory frame where the peak intensity of each Silicon Bragg peak was maximized. **Fig. 1(b)** in the main manuscript shows the crystal unit cell and crystallographic planes, which satisfy the Bragg condition for the $(2\bar{2}0)$ and $(1\bar{1}\bar{1})$ reflections. Each $\mathbf{Q}^{\mathrm{lab}}_{1\bar{1}\bar{1}}$ and $\mathbf{Q}^{\mathrm{lab}}_{2\bar{2}0}$ vector is perpendicular to the indicated family of atomic planes when the Bragg condition is satisfied for one set of planes, thus, during two different and independent measurements. The x-ray beam is propagating along the z axis, with the y axis pointing up, and the x axis pointing outward from the storage ring.

Then, we constructed orthonormal vector bases in the three different reference frames and derived the matrices that transform a vector expressed in one reference frame to another (the derivation of transformation matrix is given in Appendix B). In three-dimensional space the term *orthonormal* refers to any set of unitary vectors, which are normal to each other (Koks, 2006). A set of orthonormal, thus, linearly independent vectors compose a vector *basis*. Any arbitrary vector can be expressed as a linear combination of the base vectors.

For each Bragg reflection, we can calculate the $\mathbf{Q}^{\mathrm{l}ab}_{1\bar{1}\bar{1}}$, $\mathbf{Q}^{\mathrm{l}ab}_{2\bar{2}0}$ vectors, which are given by

$$\mathbf{Q}^{\mathrm{l}ab}_{hk\ell} = \mathbf{k}_f - \mathbf{k}_i. \quad (1)$$

where

$$\mathbf{k}_i = \begin{pmatrix} 0 \\ 0 \\ 1 \end{pmatrix} \quad (2)$$

since the incident x-rays are parallel to the z axis of the laboratory frame and

$$\mathbf{k}_f = \frac{2\pi}{\lambda}\begin{pmatrix} \cos\gamma\sin\delta \\ \sin\gamma \\ \cos\gamma\cos\delta \end{pmatrix} \quad (3)$$

with δ, γ the detector angles as depicted in **Fig. 1(a)** of the main manuscript and λ the x-ray wavelength.

Since we also know the Miller indices of the two measured Bragg peaks $(1\bar{1}\bar{1})$, $(2\bar{2}0)$, we can calculate the $\mathbf{Q}^{cryst}_{1\bar{1}\bar{1}}$ and $\mathbf{Q}^{cryst}_{2\bar{2}0}$ vectors in the crystal frame (Zhang *et al.*, 2014):

$$\mathbf{Q}^{cryst}_{hk\ell} = \frac{[h,k,\ell]}{\parallel [h,k,\ell] \parallel} = \frac{[h,k,\ell]}{\sqrt{h^2+k^2+\ell^2}} \quad (4)$$

We used the $\mathbf{a} \equiv \mathbf{Q}^{cryst}_{1\bar{1}\bar{1}}$ and $\mathbf{b} \equiv \mathbf{Q}^{cryst}_{2\bar{2}0}$ vectors shown in **Fig. S1** to construct an orthogonal vector basis in the crystal frame $\{\hat{\mathbf{e}}^c_i\}$: $\{\hat{\mathbf{e}}^c_1, \hat{\mathbf{e}}^c_2, \hat{\mathbf{e}}^c_3\}$ with all vectors being unitary. First, we project vector $\mathbf{b}$ on $\mathbf{a}$. This gives us a new vector

$$\mathbf{a}_1 = \parallel \mathbf{b} \parallel \cos\omega\hat{\mathbf{a}} = \parallel \mathbf{b} \parallel \frac{\mathbf{a}\cdot\mathbf{b}}{\parallel \mathbf{a} \parallel\parallel \mathbf{b} \parallel}\hat{\mathbf{a}} = (\mathbf{b}\cdot\hat{\mathbf{a}})\hat{\mathbf{a}} \quad (5)$$

with ω the angle between the two Bragg reflections (vectors $\mathbf{a}, \mathbf{b}$), which is 35.26° between the $[111]$ and $[110]$ families of atomic planes. The difference between the $\mathbf{b}$ and $\mathbf{a}_1$ vectors correspond to a vector, which is perpendicular to the $\mathbf{a}$ and $\mathbf{a}_1$ vectors as shown in **Fig. S1**.

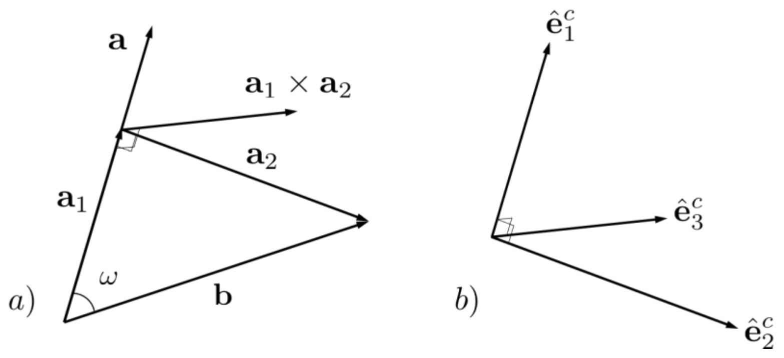

Figure S1 Construction of a vector basis from two known vectors.

We pick the third linearly independent vector to be the cross product of the two vectors. Thus, we have

$$\mathbf{a}_2 = \mathbf{b} - \mathbf{a}_1 \quad \text{and} \quad \mathbf{a}_3 = \mathbf{a}_1 \times \mathbf{a}_2 \qquad (6)$$

Then, by normalizing the $\mathbf{a}_1, \mathbf{a}_2, \mathbf{a}_3$ vectors we create a vector basis in the crystal frame, shown in **Fig. S1(b)**.

$$\{\hat{\mathbf{e}}_i^c\}: \{\hat{\mathbf{e}}_1^c, \hat{\mathbf{e}}_2^c, \hat{\mathbf{e}}_3^c\} \qquad (7)$$

Similarly, we can construct from the measured $\mathbf{Q}_{1\bar{1}\bar{1}}^{\ell ab}$ and $\mathbf{Q}_{2\bar{2}0}^{\ell ab}$ vectors a vector basis in the laboratory frame

$$\{\hat{\mathbf{e}}_\ell\}: \{\hat{\mathbf{e}}_1^\ell, \hat{\mathbf{e}}_2^\ell, \hat{\mathbf{e}}_3^\ell\}. \qquad (8)$$

Consider A a transformation matrix, which transforms a vector basis in the crystal frame to the laboratory frame such

$$A\hat{\boldsymbol{e}}_{\boldsymbol{c}} = \hat{\boldsymbol{e}}_{\boldsymbol{\ell}} \Rightarrow A[\hat{\boldsymbol{e}}_1^c \hat{\boldsymbol{e}}_2^c \hat{\boldsymbol{e}}_3^c] = [\hat{\boldsymbol{e}}_1^\ell \hat{\boldsymbol{e}}_2^\ell \hat{\boldsymbol{e}}_3^\ell]$$

$$\Rightarrow \quad A = [\hat{\boldsymbol{e}}_1^\ell \hat{\boldsymbol{e}}_2^\ell \hat{\boldsymbol{e}}_3^\ell][\hat{\boldsymbol{e}}_1^c \hat{\boldsymbol{e}}_2^c \hat{\boldsymbol{e}}_3^c]^{-1} \qquad (9)$$

B, a matrix that transforms a vector basis in the crystal frame to the sample frame

$$B\hat{\boldsymbol{e}}_{\boldsymbol{c}} = \hat{\boldsymbol{e}}_{\boldsymbol{s}} \Rightarrow B[\hat{\boldsymbol{e}}_1^c \hat{\boldsymbol{e}}_2^c \hat{\boldsymbol{e}}_3^c] = [\hat{\boldsymbol{e}}_1^s \hat{\boldsymbol{e}}_2^s \hat{\boldsymbol{e}}_3^s]$$

$$\Rightarrow \quad B = [\hat{\boldsymbol{e}}_1^s \hat{\boldsymbol{e}}_2^s \hat{\boldsymbol{e}}_3^s][\hat{\boldsymbol{e}}_1^c \hat{\boldsymbol{e}}_2^c \hat{\boldsymbol{e}}_3^c]^{-1} \qquad (10)$$

and C, a matrix transforming a vector basis in the sample frame to the laboratory frame

$$C\hat{\boldsymbol{e}}_{\boldsymbol{s}} = \hat{\boldsymbol{e}}_{\boldsymbol{l}} \Rightarrow C[\hat{\boldsymbol{e}}_1^s \hat{\boldsymbol{e}}_2^s \hat{\boldsymbol{e}}_3^s] = [\hat{\boldsymbol{e}}_1^l \hat{\boldsymbol{e}}_2^l \hat{\boldsymbol{e}}_3^l]$$

$$\Rightarrow \quad C = [\hat{\boldsymbol{e}}_1^l \hat{\boldsymbol{e}}_2^l \hat{\boldsymbol{e}}_3^l][\hat{\boldsymbol{e}}_1^s \hat{\boldsymbol{e}}_2^s \hat{\boldsymbol{e}}_3^s]^{-1} \qquad (11)$$

We can easily derive the following relations between the A, B, and C matrices:

$$\begin{aligned} \hat{\boldsymbol{e}}_{\boldsymbol{l}} &= C\hat{\boldsymbol{e}}_{\boldsymbol{s}} = CB\hat{\boldsymbol{e}}_{\boldsymbol{c}} \\ \hat{\boldsymbol{e}}_{\boldsymbol{l}} &= A\hat{\boldsymbol{e}}_{\boldsymbol{c}} \\ \Rightarrow \quad A &= CB = B^T C^T \qquad (12) \end{aligned}$$

and

$$\begin{aligned} \hat{\boldsymbol{e}}_{\boldsymbol{c}} &= A^T\hat{\boldsymbol{e}}_{\boldsymbol{l}} = A^T C\hat{\boldsymbol{e}}_{\boldsymbol{s}} \\ \hat{\boldsymbol{e}}_{\boldsymbol{c}} &= B^T\hat{\boldsymbol{e}}_{\boldsymbol{s}} \\ \Rightarrow B^T &= A^T C \Rightarrow AB^T = C \qquad (13) \end{aligned}$$

The transformation matrix B translates the crystal orientation in the crystal frame to an orientation in the sample frame, which can be described in principle, by two in-plane and out-of-plane vectors. In the laboratory frame, by definition $\theta = 0, \chi = 90°$, and $\phi = 0$. Thus, to simulate any Laue pattern, which is acquired at a specific set of goniometer angles one must consider the rotation of the sample to that set of angles. The determination of rotation matrix from a set of goniometer angles is mentioned Appendix B. The incident beam is parallel to z_ℓ, y_ℓ is up and x_ℓ chosen to form a right-hand system. At the APS, x_ℓ points outwards of the experimental hutch, $z_\ell = x_\ell \times y_\ell$. The sample frame provides a basis in which to represent a uniquely oriented crystal specimen. For an arbitrarily oriented sample, coordinates in $\{x_s, y_s, z_s\}$ are related to $\{x_\ell, y_\ell, z_\ell\}$ via the rotation matrix (the sample frame w.r.t. the Lab frame) :

$$\mathbf{G} = \mathbf{R}_{\theta,y}\mathbf{R}_{90-\chi,z}\mathbf{R}_{\phi,x} \qquad (14)$$

Then, we need to define the conventional Bunge-style orientation matrix for the crystal with respect to the sample reference frame. If we have a set of Bunge Euler angles (in our case, single-crystal (001)-oriented Silicon is $\phi_1 = 0, \Phi = 0, \phi_2 = 0$), we can obtain the orientation matrix $\mathbf{g_{ij}}$ in Appendix B. With the knowledge of two sets of goniometer angles $\theta_1, \chi_1, \phi_1, \theta_2, \chi_2, \phi_2$, we can get the rotation matrix $\mathbf{G}_1$ and $\mathbf{G}_2$ by Equation 14. We can apply the rotation matrix $\mathbf{G}_1$ and $\mathbf{G}_2$ and the orientation matrix $\mathbf{g_{ij}}$ to the corresponding $\mathbf{Q}^{\mathrm{l}ab}_{2\bar{2}0}$, $\mathbf{Q}^{\mathrm{l}ab}_{1\bar{1}\bar{1}}$, which relates the vectors in the lab frame (e.g., a diffracted beam arriving on the Bragg detector, or on the Laue detector) to crystal directions.

$$\mathbf{Q}^{cryst}_{2\bar{2}0} = \mathbf{g}_{ij} \cdot \mathbf{Q}^{\ell ab}_{2\bar{2}0} \cdot \mathbf{G}_1 \qquad (15)$$

$$\mathbf{Q}^{cryst}_{1\bar{1}\bar{1}} = \mathbf{g}_{ij} \cdot \mathbf{Q}^{\mathrm{l}ab}_{1\bar{1}\bar{1}} \cdot \mathbf{G}_2 \qquad (16)$$

$$\theta = \arccos\frac{\mathbf{Q}^{\mathrm{cryst}}_{1\bar{1}\bar{1}}\mathbf{Q}^{\mathrm{cryst}}_{2\bar{2}0}}{\parallel \mathbf{Q}^{\mathrm{cryst}}_{1\bar{1}\bar{1}}\mathbf{Q}^{\mathrm{cryst}}_{2\bar{2}0} \parallel} \qquad (17)$$

Combining **Error! Reference source not found.**, **Error! Reference source not found.**, and **Error! Reference source not found.**, we can fully constrain the orientation of the crystal with respect to the lab frame. This

method determines the orientation of the substrate in the lab reference frame. In order to simulate the Laue pattern on the detector, we not only need the orientation matrix of substrate in the lab frame, but also the translation vector $\mathbf{P}$ and the rotation matrix $\mathbf{R}$ for the detector in the lab frame.

We define the vector $\mathbf{D}$ as a conversion vector, coordinates in $\{x_d, y_d, z_d\}$ are connected to $\{x_\ell, y_\ell, z_\ell\}$ via the rotation matrix $\mathbf{R}$ and translation vector $\mathbf{P}$. we use the following equation to calculate the coordinates of $\mathbf{D}$ on the detector:

$$\mathbf{D} = \mathbf{R}[(x_\ell, y_\ell, z_\ell) + \mathbf{P}] \qquad (18)$$

The rotation matrix $\mathbf{R}$ can be defined in a similar way

$$\mathbf{R} = \mathbf{R}_{\delta,y}\mathbf{R}_{-\gamma,x} \qquad (19)$$

According to **Error! Reference source not found.** and Appendix B, we get the rotation matrix $\mathbf{R}$:

$$\mathbf{R} = \begin{pmatrix} \cos\delta & -\sin\delta\sin\gamma & \sin\delta\cos\gamma \\ 0 & \cos\gamma & \sin\gamma \\ -\sin\delta & -\sin\gamma\cos\delta & \cos\delta\cos\gamma \end{pmatrix} \qquad (20)$$

Then, the detector parameters will be optimized by the methods in Appendix C.

S2. Mathematical determination of rotation/orientation matrix from a set of specific set of goniometer angles

The lab frame is intended to provide a global reference frame that is stationary during the experiment. With regards to the geometric construction, we define the incident beam to be parallel to z_ℓ, y_ℓ is up and x_ℓ chosen to form a right-hand system (at the APS, x_ℓ points out the door of the hutch), $z_\ell = x_\ell \times y_\ell$. The sample frame provides a basis in which to represent a uniquely oriented and located specimen. For the arbitrarily defined sample-relative vector $\vec{S}$, coordinates in $\{x_s, y_s, z_s\}$ are related to $\{x_\ell, y_\ell, z_\ell\}$ via the rotation matrix $\mathbf{G} = \mathbf{R}_{\theta,y}\mathbf{R}_{90-\chi,z}\mathbf{R}_{\phi,x}$ in Equation 14.

$$\mathbf{R}_{\theta,y} = \begin{Bmatrix} cos\theta & 0 & sin\theta \\ 0 & 1 & 0 \\ -sin\theta & 0 & cos\theta \end{Bmatrix} \quad (21a)$$

$$\mathbf{R}_{90-\chi,z} = \begin{Bmatrix} cos(90-\chi) & -sin(90-\chi) & 0 \\ sin(90-\chi) & cos(90-\chi) & 0 \\ 0 & 0 & 1 \end{Bmatrix} = \begin{Bmatrix} sin\chi & -cos\chi & 0 \\ cos\chi & sin\chi & 0 \\ 0 & 0 & 1 \end{Bmatrix} \quad (21b)$$

$$\mathbf{R}_{\phi,x} = \begin{Bmatrix} 1 & 0 & 0 \\ 0 & cos\phi & -sin\phi \\ 0 & sin\phi & cos\phi \end{Bmatrix} \quad (21c)$$

Combining Equation 14 and Equation 21, we can get the rotation matrix $\mathbf{G}$.

$$\mathbf{G} = \begin{Bmatrix} \cos\theta\sin\chi & -\cos\theta\cos\chi\cos\varphi+\sin\theta\sin\varphi & \cos\theta\cos\chi\sin\varphi+\sin\theta\cos\varphi \\ \cos\chi & \sin\chi\cos\varphi & -\sin\chi\sin\varphi \\ -\sin\theta\sin\chi & \sin\theta\cos\chi\cos\varphi+\sin\varphi\cos\theta & -\sin\theta\sin\varphi\cos\chi+\cos\theta\cos\varphi \end{Bmatrix} \quad (22)$$

Finally, we can define the conventional (Bunge-style) orientation matrix from a set of Bunge Euler angles $\{\phi_1, \Phi, \phi_2\}$. We can express the orientation matrix $\mathbf{g_{ij}}$ as:

$$\mathbf{g_{ij}} =$$

$$\begin{Bmatrix} \cos\phi_1\cos\phi_2 - \sin\phi_1\sin\phi_2\cos\Phi & \sin\phi_1\cos\phi_2 + \cos\phi_1\sin\phi_2\cos\Phi & \sin\phi_2\sin\Phi \\ -\cos\phi_1\sin\phi_2 - \sin\phi_1\cos\phi_2\cos\Phi & -\sin\Phi_1\sin\phi_2 + \cos\phi_1\cos\phi_2\cos\Phi & \cos\phi_2\sin\Phi \\ \sin\phi_1\sin\Phi & -\cos\phi_1\sin\Phi & \cos\Phi \end{Bmatrix} \quad (23)$$

S3. Detector parameter optimization

The Laue detector parameters include the rotation vector $\mathbf{R}$ and translation vector $\mathbf{P}$, both of which contain three degrees of freedom. The translation vector $\mathbf{P}$ gives the distance between the center of the detector and the origin in the laboratory frame. The rotation vector $\mathbf{R}$ is the Rodriguez rotation vector, which rotates the detector from the origin to its actual location during the measurement. We determined the translation vector to be $\mathbf{P} = [0, -25, -65]$. The rotation vector $\mathbf{R}$ was calculated using the rotation matrix and the Rodriguez rotation formula (Pateras *et al.*, 2020; Koks, 2006). We determined the initial values of the rotation vector $\mathbf{R} = [2.3, 0.0, 2.2]$. The values are only a rough estimation and need to be optimized. With the orientation of the Silicon substrate and the initial $\mathbf{P}$ and $\mathbf{R}$, we used the forward-model to generate the Laue pattern shown in Figure S2 (a).

We then optimized the detector parameters by combining the Nelder-Mead algorithm with a grid search method. For the definition of the cost function, we used the forward model to simulate Laue diffraction, compared the simulation and experiment data, and calculated the average distances between the extracted peaks in the experiment data and corresponding nearest peak positions in the forward model. Providing the segmentation result from the experimentally measured Laue pattern of the substrate, we could locate the center-of-mass for each peak. We created a list of Miller indices for each experimentally measured Bragg peak and then found the nearest peak predicted by the forward model. The cost function calculates the average distance between the experimentally measured Bragg peaks and the corresponding nearest peaks predicted by the forward model.

Although Nelder-Mead tends to optimize the objective function fairly fast, within tens of iterations, it can be trapped in valleys around local minima. Considering this, we decided to set a few initial points for the optimization. More specifically, we picked three different values as the initial point for each of the three components of the translation vector P_0. We were able to find the optimal value of the P_0 component of the translation vector and with the same procedure determined the values of the other components of the translation vector $\mathbf{P}$ and rotation vector $\mathbf{R}$. Combining Nelder-Mead with a grid search method can solve the problem of multi-variable optimization, shown before and after in Figure S2. The values of all six components of the two vectors are listed in Table S1.

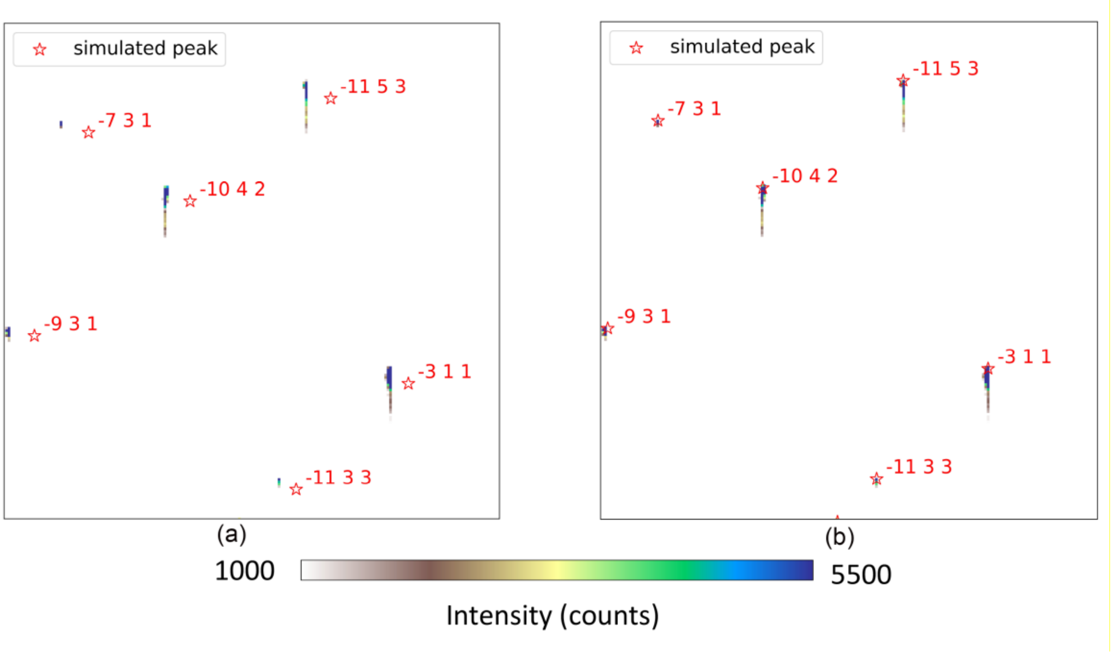

Figure S2 (a) A Laue diffraction pattern from silicon substrate and simulated Laue diffraction before detector parameter optimization; (b) The same diffraction pattern and simulated Laue diffraction after detector parameter optimization.

Table S1 Detector parameters before and after optimization

	Before Optimization	After Optimization
R [radians]	$[2.3, 0.0, 2.2]$	$[2.23, -0.009, 2.205]$
P [mm]	$[0, -25, -65]$	$[-0.653, -25.946, -68.220]$

S4. Demonstration of the twiddle algorithm on parameter optimization for rolling ball algorithm

Twiddle algorithm (https://martin-thoma.com/twiddle/) is used to search for the parameter in rolling ball algorithm (RB). A twiddle algorithm is a generic algorithm for optimizing loss functions for single values. Compared with gradient descent method, it is superior regarding to the time complexity, because of no calculation requirement for the second order derivative.

The input of twiddle algorithm is an objective function $f(x)$ that calculates the gradient checkpoint at x (derivative $\partial F/\partial r$ in 2.3 Image processing). At the beginning of twiddle refinement, we run RB algorithm with x_0 (the initial point for optimizing): $f(x)^* = f(x_0)$. We set range x_{range} for the radius, and the ε as tolerance to determine the convergence of the gradient. Then we add initial dx (increment of radius) to x, and re-calculate the objective function $f(x)$; if there was some improvement ($f(x) < f(x)^*$), then we increase the increment of radius $dx *= 1.1$ and update the optimal function value $f(x)^* = f(x)$. Otherwise, we try to decrease the radius x, and this means $x -= 2 * dx$, recalculate the $f(x)$ and compare $f(x)$ and $f(x)^*$ again. If there is some improvement, then this means we should decrease x. Thus, we adaptively changed the decrement ($dx *= 1.05$) based on the performance of new parameters. If there is no improvement, this means neither increment nor decrement improves the searching, the step size might simply be too big. Therefore, we adjust the extent of increment/decrement $dx *= 0.95$ and iteratively run the same procedure. The whole process terminates only when dx reaches a set threshold (usually 0.1) or radius x is out of range x_{range} (usually 10 pixels ~ 150 pixels).

Input: objective function $f(x)$ that calculates the gradient checkpoint at x, x_0 is the initial point for optimizing, $x_{\textbf{range}}$ is the range of x, and the ϵ is tolerance to determine the convergence of the gradient.

Output: optimal x

initial $dx = 10$;
$f(x)^* = f(x_0)$;
while $dx > \epsilon$ **and** $x \in x_{\textbf{range}}$ **do**
    $x \mathrel{+}= dx$;
    $f(x)$;
    if $f(x) < f(x)^*$ **(there was some improvement) then**
        $f(x)^* = f(x)$;
        $dx \mathrel{*}= 1.1$
    else
        $x \mathrel{-}= 2 * dx$ **(there was no improvement, go into the other direction)**;
        $f(x)$;
        if $f(x) < f(x)^*$ **(there was some improvement) then**
            $f(x)^* = f(x)$;
            $dx \mathrel{*}= 1.05$
        else
            $x \mathrel{+}= dx$ **(As there was no improvement in either direction, the step size might simply be too big)**;
            $dx \mathrel{*}= 0.95$
return x

Figure S3 Pseudo-code of twiddle algorithm for parameter optimization.

S5. Grain size distribution

The grain size distribution is generated by MTEX (https://mtex-toolbox.github.io/ShapeParameters.html). The input is sample EBSD data set and we remove all not indexed pixels and reconstruct grains followed by computing the number of pixels that belong to a certain grain. The grain area represents the actual area measured in μm^2. We analyze the distribution of grains by grain area using a histogram plotted in Figure S4.

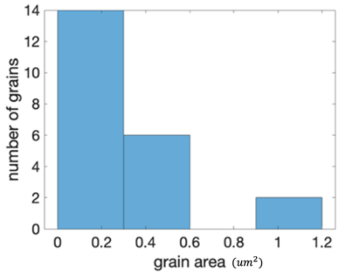

Figure S4 Grain sizes of Au crystals (unit of grain area: μm^2).

S6. An example of using rolling ball algorithm with different radius

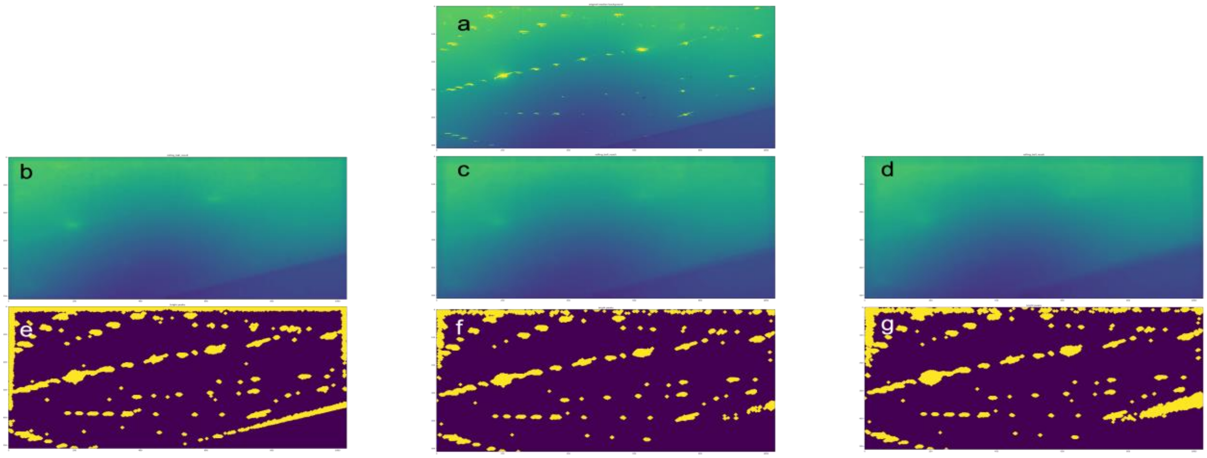

Figure S5 a: A raw image. b: The background image generated by rolling ball algorithm with radius 30. e. The binary image after background (b) subtraction, and yellow points indicate substrate peaks. c: The background image generated by rolling ball algorithm with radius 90. f. The binary image after background (c) subtraction, and yellow points indicate substrate peaks. d: The background image generated by rolling ball algorithm with radius 150. g. The binary image after background (d) subtraction, and yellow points indicate substrate peaks. Comparing Figure S5 e,f and g, radius 90 is the best because all substrate peaks are included and segmented with less false positive peaks.

S7. High-resolution SEM images for crystals

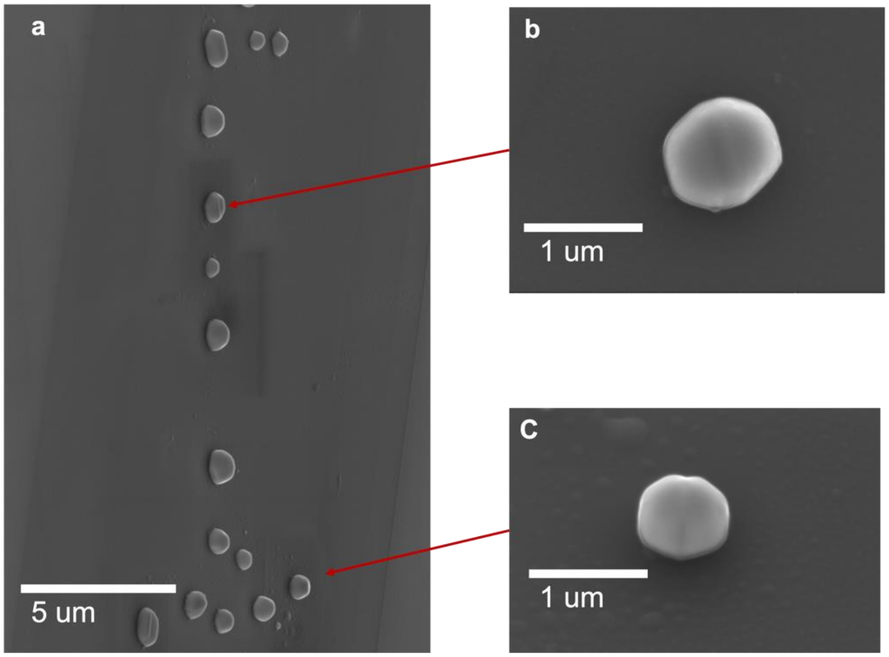

Figure S6 (a) A SEM image of the patch of crystals scanned by pink beam. (b) The crystal at the middle of this patch. (c) The crystal at the right of this patch.

S8. Determination of the radius parameter of RBA

The ball radius is the only algorithmic parameter in RBA but its value depends on the background distribution found in each experimentally acquired image. The ball radius value controls which features are left out and which are kept. Accordingly, a routine for optimizing the RBA radius for each acquired Laue pattern was established and is described below. The radius is selected to be appreciably wider than any of the apparent diffraction spots that resemble and could be falsely classified as Bragg peaks. However, the ball radius is small enough to follow the smoothly varying background and correctly subtract it. The twiddle algorithm, **Appendix D** (Thoma, 2014), is used to find the best RBA radius. The objective or cost function was chosen to be the derivative of the fraction of pixels whose intensities are below a threshold value in the corrected image (raw image with background subtracted) with respect to the tested radius. When the radius value is too small, the derivative $\partial F/\partial r$ is large because increasing the radius rapidly increases the number of peaks. According to the distribution of pixel intensities, most false positives are low in intensity. When the radius is less than two pixels, the rolling ball enters the interior of all false positives. In this situation, a small increase in radius will lead the ball to fail to enter a large number of low-intensity false positives. Therefore, there is a rapid increase of pixels classified as peaks with intensities over 10 counts/pixel in the corrected image. The derivative $\partial F/\partial r$ reaches its minimum point where the substrate peaks, each consisting of many high-intensity pixels, are included as peaks. At this point, further increases in radius do not rapidly increase the number of peaks found. Thus, more peaks are found that

are false positives (with respect to nanocrystal peaks) and the derivative increases. Thus, there is a plateau (minimum) in the number of peaks with respect to the radius that allows the objective function to identify the best value.

The initial value of radius was 20 and the allowed range of radii values is [10, 200]. The tolerance (the number used to determine when optimization has converged to a sufficiently good score) is 1 pixel. Finally, the best radius returned by the optimization for the current dataset was 90 pixels. To justify that 90 is a reasonable number, Fig. 4(b) shows the number of pixels whose intensities are above a threshold value in the corrected image with radii ranging from 0.1 to 300 pixels. The fraction of pixels F whose intensity is larger than the threshold value 10 and the derivative ∂F⁄∂r of the fraction of pixels with respect to the tested radius is calculated and shown in Fig. 4(b). The substrate peaks were then segmented from the residual noise through a global threshold τ whose value was determined by minimizing ∂F⁄∂r, as in the RBA radius optimization. Using a cluster identification algorithm (Virtanen et al., 2020), the location of all intensity clusters (substrate peaks) was identified.

The final values obtained from different initial guesses are not consistent. As an illustration, we compared the outcomes determined from varying initial values of P_2, where the initial value ranges from -20 to -30 mm in increments of 2. For each initial value, we generated one Laue pattern and chose ten Bragg peaks from each pattern. We compared these peaks with experimentally measured peaks and then computed the average distance between the extracted and corresponding nearest peak positions provided by the forward model for each Laue pattern. This average distance is known as the loss, which can be optimized using the Nelder-Mead algorithm.

S9. Loss and a standard deviation for the results determined from different starting guesses for P_2.

The final values obtained from different initial guesses are not consistent. As an illustration, we compared the outcomes determined from varying initial values of P_2, where the initial value ranges from -20 to -30 mm in increments of 2. For each initial value, we generated one Laue pattern and chose ten Bragg peaks from each pattern. We compared these peaks with experimentally measured peaks and then computed the average distance between the extracted and corresponding nearest peak positions provided by the forward model for each Laue pattern. This average distance is known as the loss, which can be optimized using the Nelder-Mead algorithm.

The subsequent table displays the loss for the results obtained from different starting guesses for P_2, and the optimized values of P_1, P_2, and P_3 for the corresponding initial guesses. The variance (σ^2) can be calculated using the following equation, where σ is the standard deviation.

$$\sigma^2 = \frac{1}{N}\sum_{i=1}^{N}(x_i - \bar{x})^2 = 0.678 \Rightarrow \sigma = 0.823$$

Here each of the independent observations x_i is the average distance calculated from the ten peaks of one Laue pattern defined as loss, and $\bar{x}$ is the average loss value for the six Laue patterns (N=6). Thus, for the tabulated values below we have an average loss value of 1.4 ± 0.8 pixels.

P_2 (initial point)	-20	-22	-24	-26	-28	-30
P_1	-0.613	-0.588	-0.596	-0.617	-0.614	-0.626
P_2	-21.548	-23.282	-25.685	-26.390	-27.426	-28.972
P_3	-65.972	-65.777	-68.444	-65.870	-66.271	-69.133
Loss	2.745	1.724	0.128	0.829	1.182	1.799

S10. Pole figures of crystals from EBSD and Laue

In order to illustrate the randomness of the misorientations, we show multiple examples of combined pole figures (PFs) of different crystals from EBSD (blue points in the figure below) and Laue (orange points in the figure below) and find that the discrepancy of orientations (misorientations) from the two analysis lacks any systematic pattern, i.e., there is no systematic offset between the Laue and EBSD measurements of orientation. We plot and show all 19 PFs below for illustration.

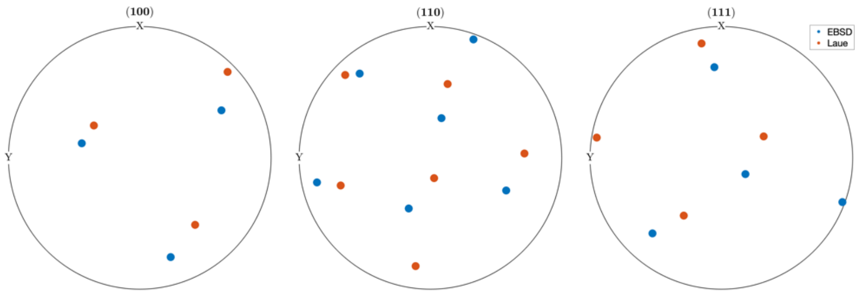

Figure S7 Pole figures from one grain (Cryst.ID = 1).

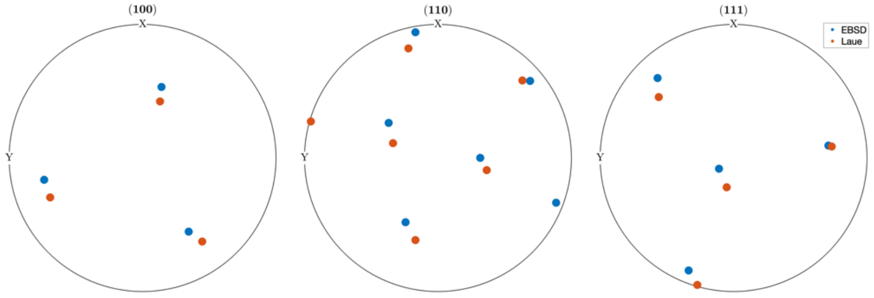

Figure S8 Pole figures from one grain (Cryst.ID = 2).

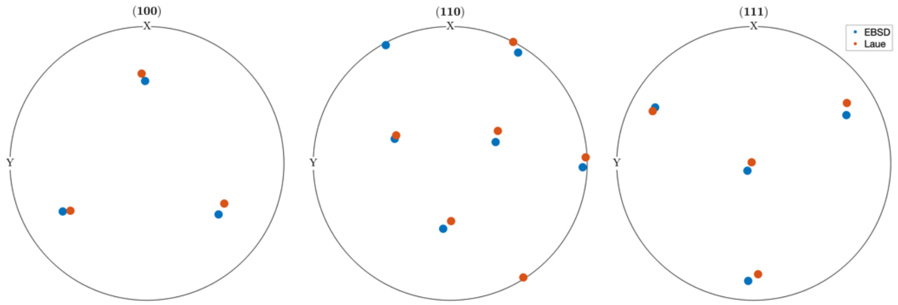

Figure S9 Pole figures from one grain (Cryst.ID = 3).

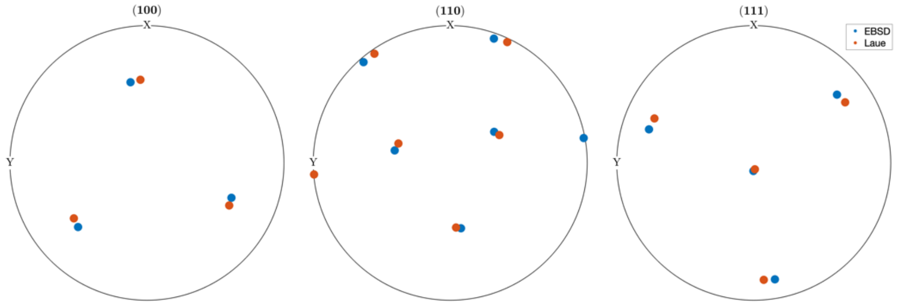

Figure S10 Pole figures from one grain (Cryst.ID = 4).

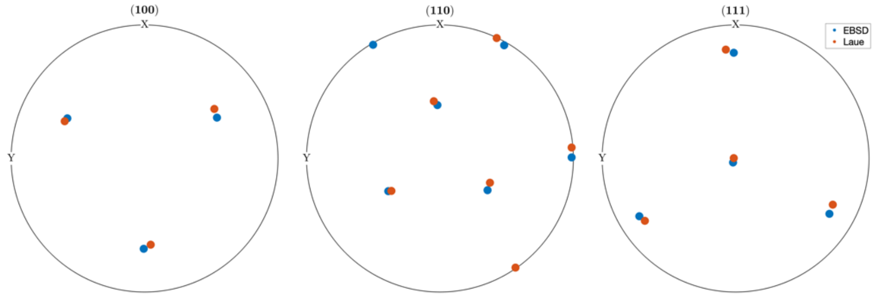

Figure S11 Pole figures from one grain (Cryst.ID = 5).

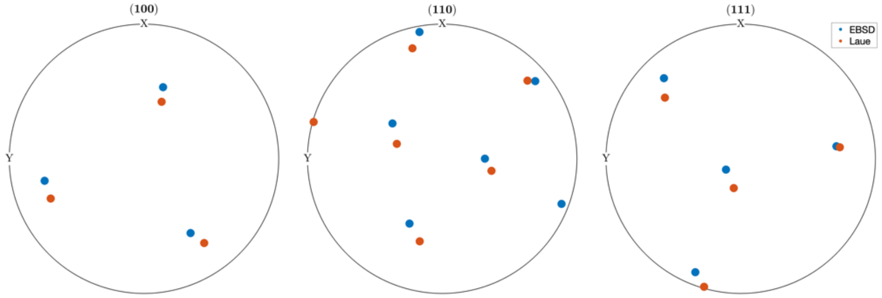

Figure S12 Pole figures from one grain (Cryst.ID = 6).

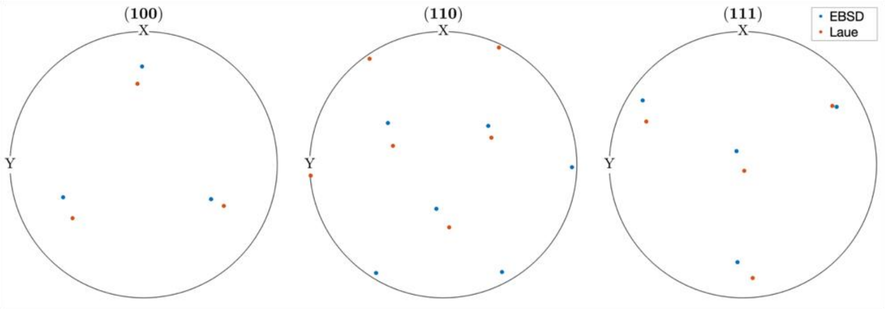

Figure S13 Pole figures from one grain (Cryst.ID = 7).

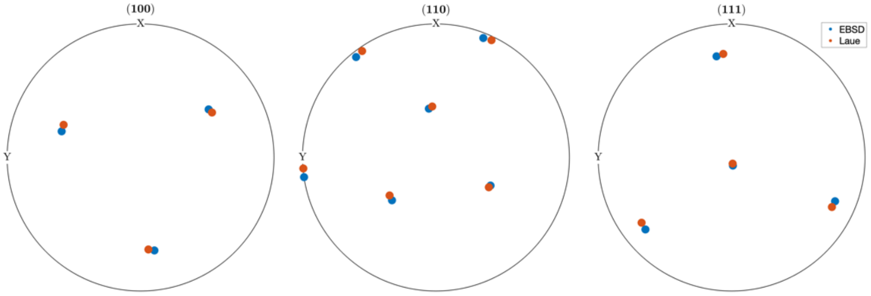

Figure S14 Pole figures from one grain (Cryst.ID = 8).

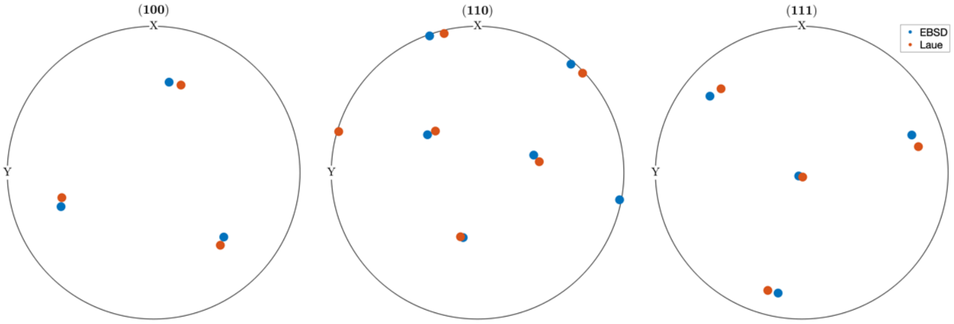

Figure S15 Pole figures from one grain (Cryst.ID = 9).

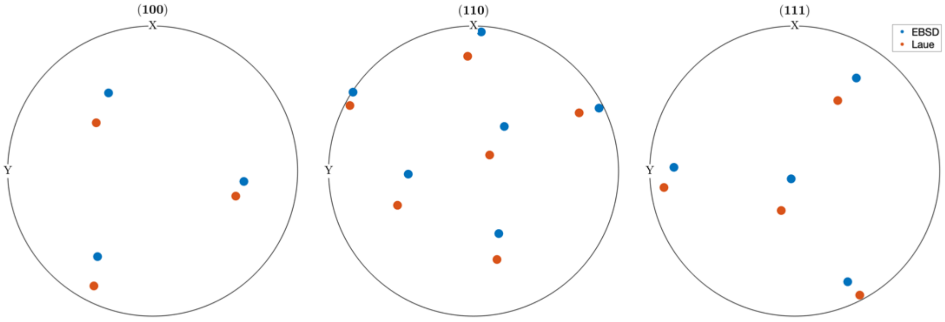

Figure S16 Pole figures from one grain (Cryst.ID = 10).

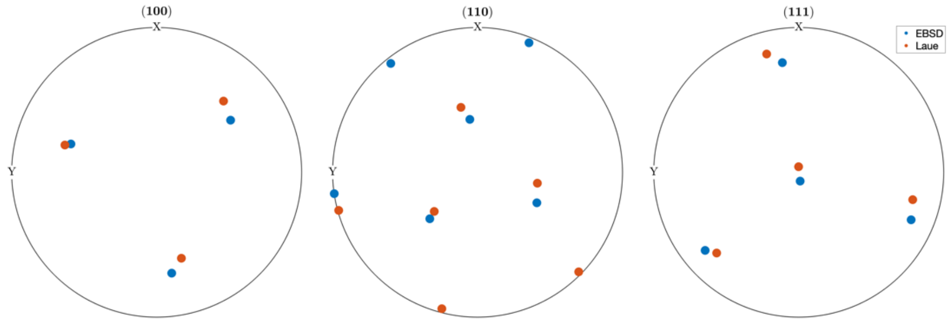

Figure S17 Pole figures from one grain (Cryst.ID = 11).

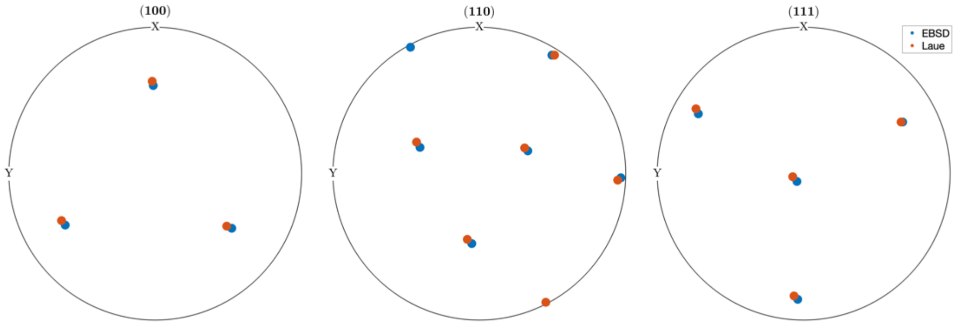

Figure S18 Pole figures from one grain (Cryst.ID = 12).

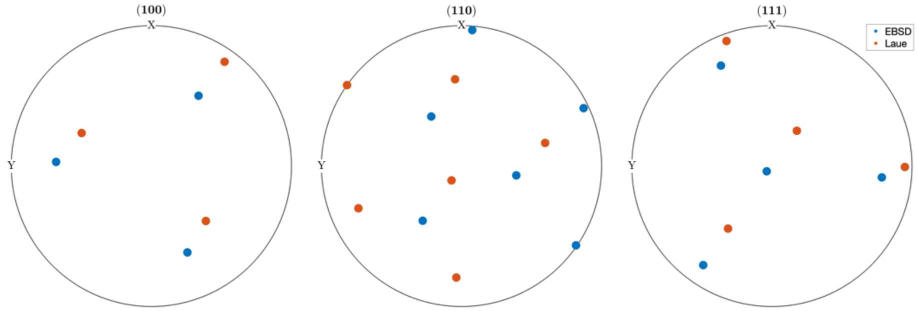

Figure S19 Pole figures from one grain (Cryst.ID = 13).

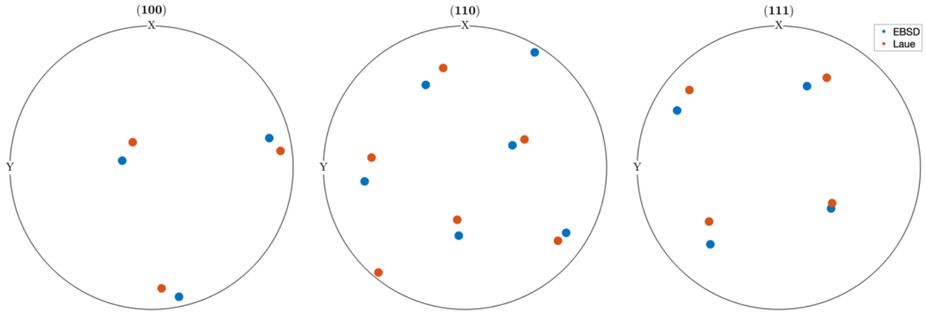

Figure S20 Pole figures from one grain (Cryst.ID = 14).

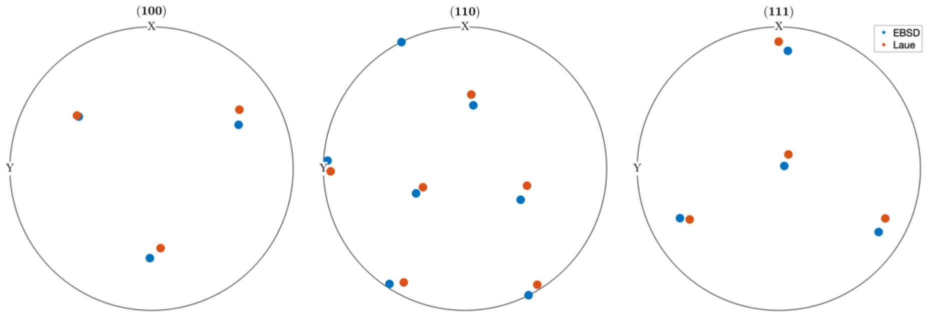

Figure S21 Pole figures from one grain (Cryst.ID = 15).

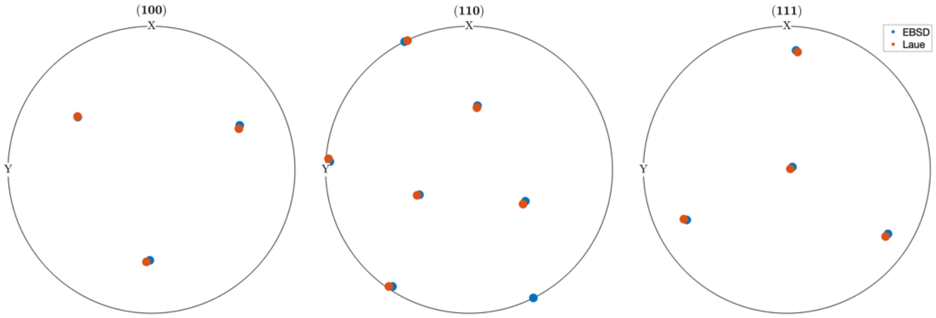

Figure S22 Pole figures from one grain (Cryst.ID = 16).

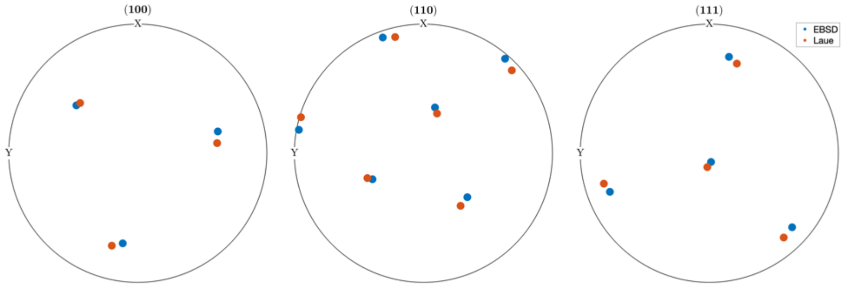

Figure S23 Pole figures from one grain (Cryst.ID = 17).

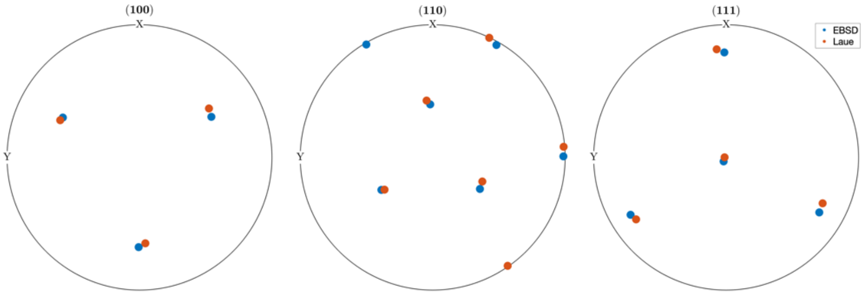

Figure S24 Pole figures from one grain (Cryst.ID = 18).

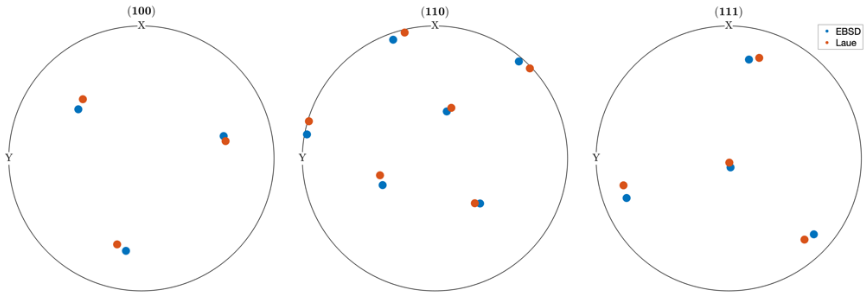

Figure S25 Pole figures from one grain (Cryst.ID = 19)
